# Emerging electrochemical energy conversion and storage technologies

**DOI:** 10.3389/fchem.2014.00079

**Published:** 2014-09-24

**Authors:** Sukhvinder P. S. Badwal, Sarbjit S. Giddey, Christopher Munnings, Anand I. Bhatt, Anthony F. Hollenkamp

**Affiliations:** Commonwealth Scientific and Industrial Research Organisation (CSIRO), Energy Flagship, Clayton SouthVIC, Australia

**Keywords:** energy, electrochemical energy systems, energy conversion, energy storage, batteries, fuel cells, electrochemical reactors

## Abstract

Electrochemical cells and systems play a key role in a wide range of industry sectors. These devices are critical enabling technologies for renewable energy; energy management, conservation, and storage; pollution control/monitoring; and greenhouse gas reduction. A large number of electrochemical energy technologies have been developed in the past. These systems continue to be optimized in terms of cost, life time, and performance, leading to their continued expansion into existing and emerging market sectors. The more established technologies such as deep-cycle batteries and sensors are being joined by emerging technologies such as fuel cells, large format lithium-ion batteries, electrochemical reactors; ion transport membranes and supercapacitors. This growing demand (multi billion dollars) for electrochemical energy systems along with the increasing maturity of a number of technologies is having a significant effect on the global research and development effort which is increasing in both in size and depth. A number of new technologies, which will have substantial impact on the environment and the way we produce and utilize energy, are under development. This paper presents an overview of several emerging electrochemical energy technologies along with a discussion some of the key technical challenges.

## Introduction

In view of the projected global energy demand and increasing levels of greenhouse gases and pollutants (NO_x_, SO_x_, fine particulates), there is a well-established need for new energy technologies which provide clean and environmentally friendly solutions to meet end user requirements. It has been clear for decades that renewable energy sources such as wind and solar would play some role in the modern grid with predictions varying on the levels of penetration and the effect that these renewable power sources would have on the stability of national grids. The role that renewable energy will play in the future energy mix is now becoming more obvious as this sector matures. As higher levels of renewable energy are integrated into national grids a greater understanding of the effect of their intermittent nature is becoming wide spread. This can result in significant mismatch between supply and demand. In addition to the changes to the power generation infrastructure, the integration of smart meters is leading to a market where energy use can be easily measured in real time. In order to maximize profit, privatized power generators and grid suppliers are increasingly promoting the use of strong financial incentives to be levied on power users to change their electrical energy usage habits. This has led to a defined cost being associated with the previously largely invisible tasks associated with managing power generation and large distribution grids. This clear cost signal has led to increased demand for energy storage for load-leveling, peak load shaving, and providing power when the renewable energy is not available at almost every level of the power generation market from small scale domestic devices to large scale grid connected systems. In the future energy mix, electrochemical energy systems will play a key role in energy sustainability; energy conversion, conservation and storage; pollution control/monitoring; and greenhouse gas reduction. In general such systems offer high efficiencies, are modular in construction, and produce low chemical and noise pollution.

In real-life applications, the limitations of single power generation or storage technology based energy solutions are now being recognized. In many instances the requirements (e.g., response time, power capability, energy density, etc.) for energy storage technologies far exceed the performance limits of current energy technology solutions and in some instances also exceed the theoretical limits of a given technology. Thus, there is a substantial current and future (new applications) global demand for hybrid energy solutions or power sources to optimize cost, efficiency, reliability, and lifetime whilst meeting the performance requirements of the applications. In this regard many electrochemical energy technologies are expected to play a key role.

In most electrochemical energy technologies, the electrode and electrolyte materials must possess the required ionic and electronic transport properties and a great deal of research is still to be performed at a fundamental level to study and optimize the electrochemistry of candidate materials, composites, and assemblies (such as catalyst and interface designs). Practical materials must operate in a multidimensional space where optimum electrochemical properties must co-exist with secondary properties such as chemical stability, compatibility with other components (thermal expansion co-efficient, strength, toughness, etc.) and at the same time they must be amenable to be fabricated into the required shapes and forms at acceptable cost. Materials and properties need to be carefully tailored and matched to suit a technological application and the environments in which they are to be used. At higher operating temperature, these requirements are more stringent and, in fact, they become critical at temperatures above 500°C. At these temperatures, other issues, such as gas sealing, interface compatibility and stability, and the design of support structures and containment materials are as challenging to solve as the technical issue directly associated with the electrochemical cells. Many materials and system integration complexities exist and these are being resolved through investments in experimental developments and through theoretical modeling. Once these challenges are solved, the practical applications of electrochemical energy technologies are numerous.

Some of the electrochemical energy technologies developed and commercialized in the past include chemical sensors for human and asset safety, energy efficiency, industrial process/quality control, and pollution control/monitoring; various types of fuel cells as clean energy devices for transport, stationary and portable power; a range of energy storage batteries; electrochemical reactors for fuel and chemical production; electrochromic smart windows for optical modulation and building efficiency; ion transport membranes for air separation; and supercapacitors (Guth et al., 2009; Scrosati et al., 2011; Yang et al., 2011; IPHE, 2012; Sbar et al., 2012; Wilson et al., 2012; Akhil et al., 2013; Carter and Wing, 2013; Harrop et al., 2014; Stiegel et al., 2014). While these technologies continue to be optimized for cost, lifetime, and performance, there is a substantial growing demand (multi billion dollars) for advanced electrochemical energy systems such as high energy density batteries for transport vehicles and stationary energy storage; next generation fuel cells with high efficiency, better performance, and long life; membrane reactors for value added chemical production; gas separation devices in medical and power generation; and hybrid fossil fuel/storage/renewable energy systems. In this paper an overview of some more recent and emerging electrochemical technologies is given and some of the fundamental challenges facing technology development are discussed.

## Hydrogen production technologies

Hydrogen is considered to be an important energy carrier and storage media for a future hydrogen economy. Hydrogen offers a sustainable energy future for both transport and stationary applications with near zero greenhouse gas emissions especially when generated by splitting water and combining with renewable energy sources (solar, wind, ocean). Since most renewable energy sources are intermittent in nature, hydrogen can act as a storage media for load leveling and peak load shaving. It can be generated when abundant renewable energy is available and stored and converted to power and heat in a fuel cell or combustion engine as per load demand based on end-use applications. A number of different electrochemical technologies are under development and these will be briefly reviewed in the following sections.

### Low temperature water electrolysis

**Figure 1:**
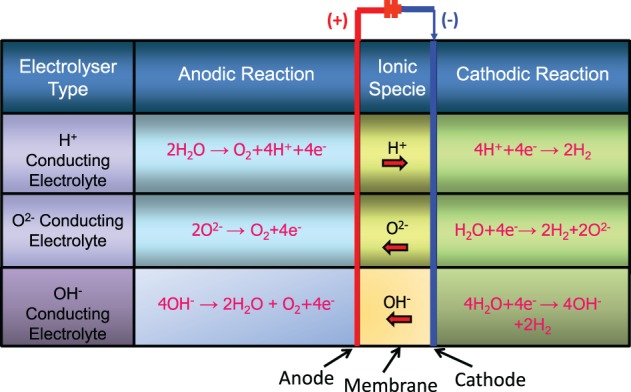
**Operating principles of low and high temperature water electrolysis with different electrolytes**.

Hydrogen can be generated by electrolyzing water at low temperatures (LTs) (<100°C) or electrolyzing steam at high temperatures (HTs) (>700–800°C). The LT electrolysis systems employ either an alkaline (hydroxyl ion conducting) solution as the electrolyte or a polymer membrane (proton conducting) as the electrolyte (Figure 1) (Ursua et al., 2012; Badwal et al., 2013). The hydrogen generation by utilizing a LT electrolyzer compared to that produced by natural gas (NG) reforming or coal gasification, offers a number of advantages such as on-site, on-demand (distributed) generation, high purity hydrogen, and unit modularity. Furthermore, such systems offer fast start-up and shutdown, and good load following capability that makes them suitable for integrating with intermittent renewable energy sources such as solar PV and wind generators. In LT systems, polymer electrolyte membrane (PEM)-based systems offer additional advantages over alkaline systems such as higher current densities (small foot print in terms of kgs per hour hydrogen generation capacity per unit stack volume), all solid state system requiring no alkaline solutions or electrolyte top-up, and higher purity hydrogen and hydrogen generation at significantly higher pressures (Badwal et al., 2013).

A typical electrolyzer system may comprise of the electrolyzer stack and balance of plant (BOP) subsystems for water deionization and circulation to anode chamber, water/gas separation (for oxygen and hydrogen), heat management, hydrogen drying and storage, and a DC power source. The stack constitutes a number of cells or membrane electrode assemblies (MEAs), assembled between bipolar metallic interconnects. The interconnects supply and collect respectively the reactants and products from cells and connects the cells in series. Further details on MEAs and electrolyzer stack assembly can be found in references (Clarke et al., 2009; Giddey et al., 2010; Ursua et al., 2012).

A number of companies (Proton OnSite, Giner Electrochemical Systems, Hydrogenics, Horizon, ITM Power) are now selling LT electrolysis systems at prices which are commercially not competitive with other processes for hydrogen production (e.g., NG steam reforming). Thus, a number of challenges related to high cost of commercial units, lifetime and net efficiency still remain.

**Figure 2:**
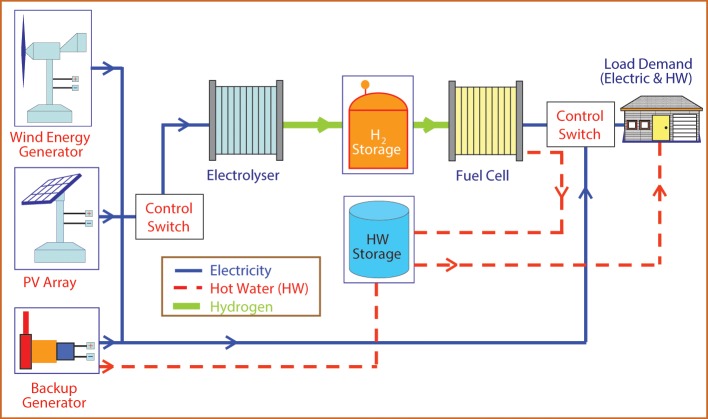
**Overall concept of a hydrogen renewable energy system for distributed power generation**.

Furthermore, the hydrogen generation by electrolysis is an energy intensive process and most commercial electrolyzers require an electric power input of 6.7–7.3 kWh/Nm^3^ (~50–55% efficiency based on HHV) of hydrogen (Badwal et al., 2013), and this increases the cost of hydrogen production and advantages of hydrogen as a clean fuel are lost if the electricity is supplied from fossil fuel resources. However, if the electric energy input can be supplied from renewable sources of energy and the electrolyzer system efficiency increased to 75–80%, then the technology becomes more attractive. The LT electrolyzers can easily operate with a large load variation and thus are highly suitable for integration with intermittent renewable energy sources. Figure 2 shows a concept of a renewable energy system based on hydrogen generation by direct coupling of an electrolyzer to solar PV and a wind generator. This type of system can be used to store hydrogen and operate a PEM fuel cell to provide power at times when renewable energy cannot meet the load demand. The other components shown in the diagram are a diesel generator as a backup, and a hot water storage tank to collect hot water from the PEM fuel cell that can be used for daily needs of a house.

The direct coupling of an electrolyzer to renewable sources of energy must ensure that there is a maximum transfer of electric energy from the renewable source to the electrolyzer to produce hydrogen. By incorporating appropriate maximum power point trackers (MPPT) and DC-DC converters to meet these requirements, a number of systems have been demonstrated in the past. However, this substantially adds to the cost and makes the renewable energy—hydrogen generation system economically less viable. Therefore, it would be beneficial if the renewable source of energy is directly coupled to the electrolyzer without any electronics or control system, and also without losing on the energy transfer to the electrolyzer. There have already been studies and demonstrations for hydrogen generation by coupling PEM-based electrolyzers to solar PV (Arriaga et al., 2007; Clarke et al., 2009) and to a wind generator (Harrison et al., 2009).

**Figure 3:**
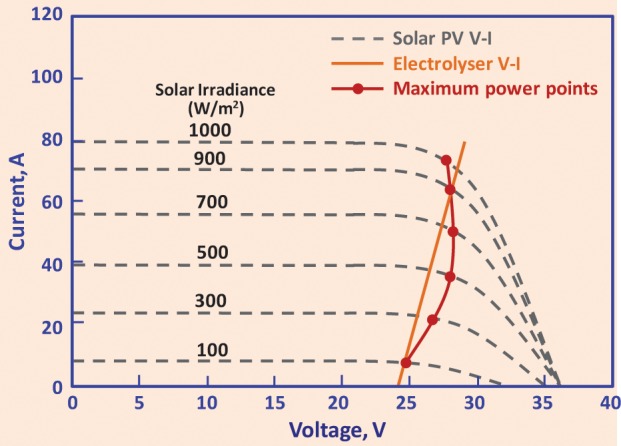
**A typical example of matching maximum power point (MPP) curve of a suitably configured solar PV array to V-I characteristics of an electrolyzer**. The example is for 15 pairs of solar PV arrays connected in parallel and a 16 cell electrolyzer. The data in the Figure has been taken from Clarke et al. (2009).

Figure 3 shows a typical example of matching the maximum power point (MPP) curve of solar PV array to the V-I characteristics of an electrolyzer (Clarke et al., 2009). The matching criteria are to achieve maximum transfer of energy from the solar PV system to the electrolyzer by matching the output of PV to the input power requirements of the electrolyzer. In the example in Figure 3, this was achieved by coupling 15 pairs of solar PV arrays in parallel to a 16 cell electrolyzer stack. The modeling of such a system showed that there will be on average 99.7% of the solar PV energy transfer to electrolyzer at all values of solar irradiance, and about 8% overall solar to hydrogen efficiency. Although the direct coupling of the renewable sources to an electrolyzer offers a relatively cheaper and more efficient way of generating hydrogen, there are two major challenges to this technology—first is on the relative sizing of the two units due to variability of the energy source (solar irradiance and wind speed) to achieve maximum benefits of coupling, and second is on the long-term performance of the electrolyzer on a continuously variable load. In a recent publication (García-Valverde et al., 2011), the authors have endeavored to tackle the first challenge by modeling polarization (V-I) curves of both, the solar PV and the electrolyzer. In relation to the second challenge, in a study carried out by NREL, a prototype electrolyzer was tested on a variable (wind generator) load profile for up to 7500 h with a small degradation in the electrolyzer performance (Harrison and Peters, 2013), however, the electrolyzer failed soon afterwards.

### High temperature water electrolysis

**Figure 4:**
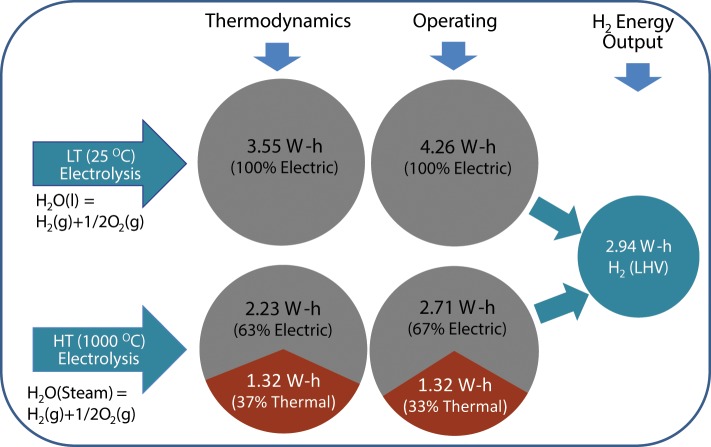
**Break down of energy input for the production of hydrogen from electrolysis at 25°C and 1000°C**. The data in the Figure has been taken from Badwal et al. (2013).

As discussed above, hydrogen can be readily produced via LT electrolysis at almost any scale using only water and electricity as the inputs. This process is well-established but requires a high input of electrical energy in order to produce the hydrogen. From a thermodynamic perspective at 25°C, 1 liter of hydrogen requires a minimum 3.55 kWh of electrical energy as an input. This increases to around 4.26 kWh when electrochemical cell losses are taken into account. If the electrolysis process is carried out at HT then it is possible to utilize some of the heat for the production of hydrogen. This contribution can be high with up to a 1/3rd of the energy required to produce the hydrogen coming from thermal energy at around 1000°C (Figure 4) (Edwards et al., 2002; Brisse et al., 2008; Laguna-Bercero, 2012; Ursua et al., 2012; Badwal et al., 2013). In Figure 4, the thermal energy input under cell operation may be slightly different due to internal heating of the cell resulting from current passage, however, due to the difficulty in making an estimate, it has been assumed to be the same as that under open circuit cell conditions. The HT electrolysis systems use an oxygen ion (O^2−^) or proton conducting (H^+^) ceramic as the electrolyte (Figure 1) (Edwards et al., 2002; Brisse et al., 2008; Laguna-Bercero, 2012; Ursua et al., 2012; Badwal et al., 2013). The process is the reverse to that of a solid oxide fuel cell (SOFC) with many similar materials used for cell construction. The thermal input required for HT systems can be supplied from different sources including renewable or sustainable energy sources or nuclear energy.

A number of different systems have been proposed including the co-locating of the electrolyzer with a solar thermal source, nuclear power stations, or supplying heat produced from the burning of low grade fuels such as coal (Edwards et al., 2002; Fujiwara et al., 2008; Badwal et al., 2013). A number of systems and materials configurations have been trialed with zirconium-based oxide ion conducting electrolytes in conjunction with manganite-based anodes and metal cermet cathodes being the most commonly used materials (Ursua et al., 2012; Badwal et al., 2013). There have been a number of reasonably significant demonstrations of this technology (up to 15 kW) but no commercial or near commercial prototypes produced (Badwal et al., 2013). These trials have demonstrated the technical feasibility of this technology, however, cost, lifetime, and reliability remain as some of the key challenges (Badwal et al., 2013). If HT electrolysis is to be commercialized then there would need to be either a significant increase in the cost of hydrocarbon fuels or a significant reduction in the cost of HT electrolyzers. The HT systems, despite offering energy efficiency advantages due to thermal input, are still at early stages of development.

In order for hydrogen to be cost competitive with other hydrocarbon fuels, the US DOE have set a cost target of $3/kg of hydrogen. If electricity and water are the only inputs (as is the case at 25°C), this leads to the electricity cost needing to be well-below 0.06 c/kWh (Badwal et al., 2013). Although this is potentially feasible, the additional costs associated with compression, transportation, and distribution make the conversion of high grade electrical power from the grid directly to hydrogen uneconomical. However, if a suitable source of thermal energy can be used then electrical component contribution reduces significantly.

### Carbon-assisted hydrogen production

The use of hydrogen as a transport fuel in fuel cell or internal combustion engine vehicles is likely to increase due to the concerns over oil shortage and rising greenhouse gas and other pollutant emissions. Hydrogen is generated mainly from NG and coal involving three major steps requiring separate reactors, all operating at temperatures in excess of 500°C: (i) NG reforming or coal gasification to produce syngas (a mixture of hydrogen and carbon monoxide) at temperatures close to 800°C; (ii) water gas shift reaction to convert carbon monoxide to hydrogen and carbon dioxide at around 500°C; and (iii) H_2_/CO_2_ separation and gas cleaning. Hydrogen production by water or steam electrolysis in which the electricity is drawn from the grid is overall a highly inefficient process, in that it requires electric input of 4.2–5 kWh per Nm^3^ and 6.7–7.3 kWh per Nm^3^ of hydrogen for the electrolysis cell stack and system, respectively.

**Figure 5:**
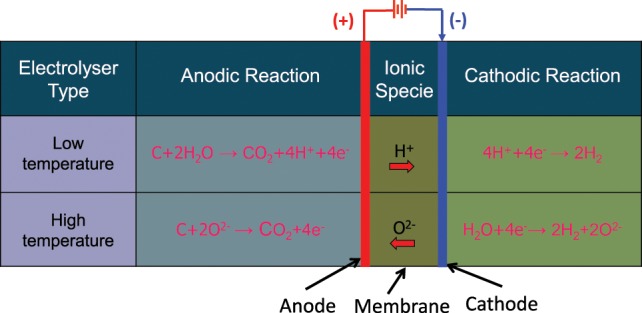
**Electrochemical reactions involved in low and high temperature carbon-assisted electrolysis process for hydrogen generation**.

The participation of carbon in the anodic reaction of the electrolysis results in a drop in the thermo-neutral voltage from 1.48 to 0.45 V required for electrolysis of water near room temperature (Coughlin and Farooque, 1982), which can translate into reduction in electric energy input to 1/3rd compared to normal electrolysis. Thus, the remaining 2/3rd of the energy would be supplied from the chemical energy of carbon. The carbon-assisted electrolysis carried out at higher temperatures can result in further reduction in the required electric energy input due to increased thermal energy contribution into the process by lowering the thermo-neutral voltage further (Seehra and Bollineni, 2009; Ewan and Adeniyi, 2013). Figure 5 schematically shows the electrochemical reactions involved for carbon-assisted electrolysis carried out at temperature <100°C (LT) employing a proton conducting electrolyte membrane, and at HTs (>800°C) employing an oxygen ion conducting ceramic electrolyte such as yttria or scandia stabilized zirconia.

In addition to a substantial reduction in the electric energy input by the involvement of carbon, this concept for hydrogen generation combines all three steps mentioned above for hydrogen from NG or coal in a single reactor. The operating temperature is expected to be low (for proton conducting electrolyte membrane used) with the overall reaction being: C + 2H_2_O → CO_2_ + 2H_2_. Furthermore, the process would generate pure hydrogen and CO_2_ in separate compartments of the electrochemical cell separated by the impervious electrolyte membrane. Thus, the substantial cost and the 20–25% energy penalty for CO_2_ capture/separation, as is the case with other routes above, can be avoided. Carbon source can be coal or biomass. All these advantages directly translate into a highly efficient process with low overall cost and substantially reduced CO_2_ emissions.

While the hydrogen generation by carbon-assisted electrolysis clearly offers significant advantages, the area is largely unexplored. Most of the investigations have been performed with sulfuric acid as the electrolyte and at temperatures below 100°C (Seehra and Bollineni, 2009; Hesenov et al., 2011; Ewan and Adeniyi, 2013). The current densities achieved are very low due to the slow carbon oxidation kinetics at LTs, and formation of films on the surface (such as illite, siderite, carbonate, etc.) of the coal particles that block the active sites on coal, thus making the reaction unsustainable (Jin and Botte, 2010). The slow kinetics of carbon participation in the electrolysis reaction requires new catalytic electrodes and electrolyte materials for optimum performance. The effect of carbon structure, purity, morphology, catalytic additives on the cell performance also requires a more detailed investigation.

A possible strategy to increase the reaction kinetics and improve the hydrogen production rates is to substantially increase the operating temperature of the carbon-assisted electrolyzer with the use of ceramic electrolytes such as doped zirconia (Figure 5). This has the added advantage that it can further reduce the electrical power requirement as discussed in the HT electrolysis section of this article. The voltage required for HT carbon-assisted electrolysis is significantly lower than that required for the PEM-based system described above with some reports showing that hydrogen can be produced even with no applied voltage (Lee et al., 2011). Although this approach could theoretically have significant advantages in terms of cost per unit hydrogen produced, research in this area is still at a very early stage with little understanding of the mechanisms involved or the stability of materials under these operating conditions (Alexander et al., 2011; Ewan and Adeniyi, 2013). If this technology is to be taken forward, a significant effort would be required to understand the fundamental science before designing a prototype device.

## Energy conversion technologies

### Fuel cells—the next generation

**Figure 6:**
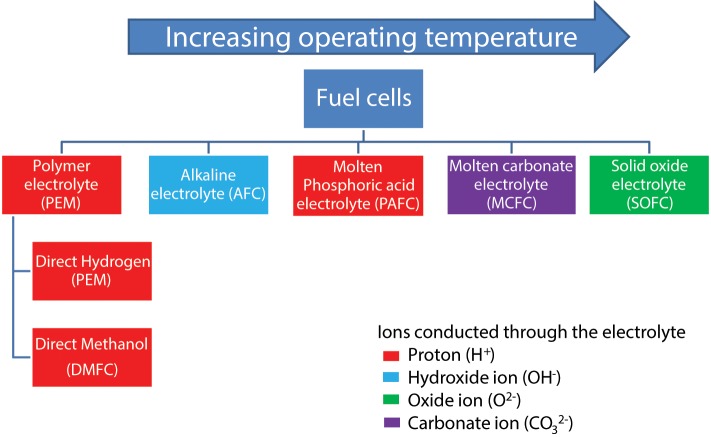
**Classification of current commercial or near commercial fuel cell systems**.

A wide variety of fuel cell systems of various scales (few W to MW range) are now commercially available and their operating regimes and widely varying performance characteristics have been discussed in the literature (Devanathan, 2008; Giddey et al., 2012; Kulkarni and Giddey, 2013; Badwal et al., 2014). These devices have traditionally been categorized firstly by the type of electrolyte and then by the type of fuel used. Fuel cells can be further categorized by the operating temperature, with polymer electrolyte membrane fuel cells (PEMFC) typically have the lowest operating temperatures below 100°C and SOFCs the highest operating around 800°C or above (Figure 6).

#### Conventional fuel cells

The operating temperature in conventional fuel cells is a critical parameter when looking at the system as it defines the type of fuel used, materials choice, end-user application, and electrical efficiency. HT systems (such as molten carbonate and SOFCs) operate at temperatures high enough to allow internal reforming of hydrocarbon fuels. This typically allows these systems to operate with total electrical efficiencies of between 45 and 60%. In contrast the LT fuel cell systems operating on hydrocarbon fuels must externally reform and clean (removing carbon monoxide) any hydrocarbon fuel used within the system. The operating temperatures of this class of fuel cells is too low to be utilized for reforming hydrocarbon fuels thus leading such systems to have lower electrical efficiencies (around 35–40% total system electrical efficiency when operated on hydrocarbon fuels) when compared to HT systems. Also the PEMFC has a very low tolerance to CO. Intermediate temperature fuel cells (typically operating between 150°C and 350°C) are in general more resilient to fuel impurities and require lower catalyst loadings. This leads to longer operating lifetimes but their electric efficiency is similar to that of LT fuel cells. If low or intermediate temperature fuel cell systems are operated directly on hydrogen, electric efficiencies greater than 50% (with system efficiency over 80% with heat recovery) can be achieved as the fuel processing losses are avoided. Table 1 compares the electrical and system efficiencies of different fuel cell systems operated on reformed hydrocarbon fuels with the values for fuel cells which directly electrochemical oxidize a fuel (Giddey et al., 2012). Any energy from the fuel that is not converted into electrical power is lost as waste heat. A detailed description of how to calculate the total efficiency of a fuel cell system can be found in the following reference (Giddey et al., 2012). In systems where the theoretical efficiency is greater than 100% the fuel cell would require heat input for continuous operation.

**Table 1:** **Theoretical electrical efficiency of fuel cells operated on various fuels with commonly reported system values**.

**Fuel cell type**	**Fuel**	**Overall reaction**	**Operating temperature (°C)**	**Theoretical efficiency (%)**	**Actual system efficiency (%)**	
				**Electric**	**Electric**	**CHP**
PEMFC	H_2_	H_2(g)_ + ^1^/_2_O_2(g)_ = H_2_O_(l)_	60–80	83	45–50	80–90
PEMFC	NG	CH_4(g)_ + 2O_2(g)_ = CO_2(g)_ + 2H_2_O_(l)_	60–80	–	35–40	80–90
DMFC	CH_3_OH	CH_3_OH_(l)_ + 1^1^/_2_O_2(g)_ = CO_2(g)_ + 2H_2_O_(l)_	20–60	97	20–25	n/a
AFC	H_2_	H_2(g)_ + ^1^/_2_O_2(g)_ = H_2_O_(l)_	70	83	45–60	n/a
PAFC	NG	CH_4(g)_ + 2O_2(g)_ = CO_2(g)_ + 2H_2_O_(g)_	200	–	40	90
SOFC	NG	CH_4(g)_ + 2O_2(g)_ = CO_2(g)_ + 2H_2_O_(g)_	600–1000	92	45–60	90
MCFC	NG	CH_4(g)_ + 2O_2(g)_ = CO_2(g)_ + 2H_2_O_(g)_	650	92	45–55	90
DCFC	Carbon	C_(s)_ + O_2(g)_ = CO_2(g)_	500–1000	100	70–80	90

*PEMFC, Polymer Electrolyte Membrane Fuel Cell; DMFC, Direct Methanol Fuel Cell; AFC, Alkaline Fuel Cell; PAFC, Phosphoric Acid Fuel Cell; SOFC, Solid Oxide Fuel Cell; MCFC, Molten Carbonate Fuel Cell; DCFC, Direct Carbon Fuel Cell*.

The maximum electric efficiency of a fuel cell system operating on a reformed fuel, in general, is significantly lower than the theoretical maximum where fuel is directly oxidized in the electrochemical reaction of the fuel. This is because all current fuel cells operate on either pure H_2_ or (at HT) a mixture of CO and H_2_. These fuels are produced, in general, via the reforming or gasification of a hydrocarbon fuel. Reforming of any readily available hydrocarbon fuel requires significant energy input. This is particularly detrimental when an external reformer and fuel processer is used (as is mostly the case for low and intermediate temperature fuel cell systems) because none of the low grade waste heat produced via the fuel cell reactions can be used for reforming. External reforming and fuel processing is a requirement for all LT systems as these systems operate significantly below the temperature required for external reforming (around 500°C). Higher temperature systems can use waste heat from the reactions within the fuel cell to reform the incoming fuel. This results in significantly higher electrical efficiencies being reported for HT commercial systems that operate in this manner (45–60%).

There are two strategies being pursued in order to further increase the efficiency of HT fuel cells operated on gaseous hydrocarbon fuels. The first is to improve the thermal coupling between the fuel cell and the reforming reactions. This is achieved in practice by reducing the physical distance between the zone where the reforming reactions occur and the fuel cells themselves with the ideal being the direct injection of the fuel into the anode chamber. This strategy has a number of technical challenges associated with the instability of hydrocarbon fuels at HTs. These fuels typically decompose to carbon (coking) on the anode surface during the HT operation. This carbon formation can be rapid and results in the fuel cell anode being irreparably damaged. It is also common for coking to occur within the pipe work leading into a fuel cell stack blocking the pipes and stopping the fuel supply to the fuel cell. Coking can be avoided if significant amounts of steam or CO_2_ can be introduced to the fuel stream, however, this will significantly reduce the efficiency of the system.

An alternative strategy is to use materials that are more resistance to coking (typically ceramic- or Cu-based anodes). If the residence time of the fuel exposed to HT can be reduced and if anode materials which do not catalyze coking reactions can be used, then it is possible to electrochemically oxidize hydrocarbon fuels directly within a fuel cell via a multi-stage process on the surface of the anode. A number of authors have reported direct oxidation of simple hydrocarbon fuels (such as CH_4_), however, the practical difficulties associated with supplying an unstable fuel directly to the reaction sites within a fuel cell have meant that this approach has never been successfully demonstrated at any significant scale (Carrette et al., 2005).

The system cost generally increases with increasing operating temperature as more expensive materials must be used within the system to withstand the harsher operating environment. Detailed reviews of the status of current high, intermediate and low temperature fuel cells are available in the references (Carrette et al., 2005; Devanathan, 2008; Giddey et al., 2012; Kulkarni and Giddey, 2013; Badwal et al., 2014).

Although fuel cell systems are becoming increasingly commercially available there are still sufficient technical challenges that need to be overcome before the mass adoption of fuel cell technology can take place. These challenges relate to lifetime, cost, and suitable fuel supply (for low or intermediate temperature systems). Significant progress is being made through careful engineering of systems to alleviate a number of the issues, including the development of new materials with longer lifetimes, development of materials to allow transport and storage of hydrogen, low cost fabrication technologies for cell and system components and miniaturized fuel processing units for use with LT fuel cells. These advancements are incrementally increasing the appeal of fuel cell systems, however, new developments are required to make the revolutionary advancements necessary to allow fuel cells to begin to displace a significant fraction of conventional power generation capacity.

There is no one fuel cell technology that stands out as being a clear leader in terms of technology maturity or technical superiority. In general the main focus is to develop more fuel flexible systems that can operate on a wider range of fuels at increased electrical efficiency. The requirement for increased efficiency is driving research and development away from systems requiring fuel pre-processing toward systems where the fuel is directly electrochemically oxidized or where the fuel is directly fed to the anode chamber within a fuel cell. This is because this allows the maximum transfer of chemical energy to electrical energy with any waste (thermal) energy from the operation being available to either maintain the operating temperature of the device or used directly in the chemical or electrochemical reactions within the fuel cell chamber. In addition, there is also an increased interest in lowering the operating temperature of fuel cells to reduce overall system cost whilst extending the life of the fuel cell.

#### Emerging fuel cell technologies

**Figure 7:**
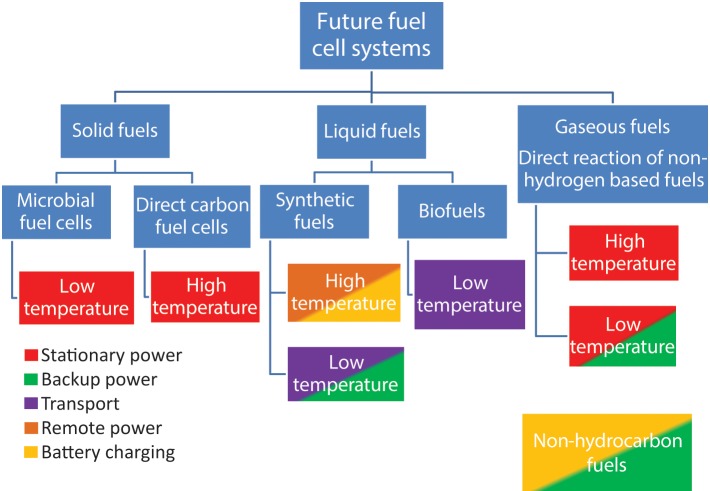
**Classification of future fuel cell systems**.

Emerging fuel cell technologies do not fit comfortably within traditional fuel cell categories in particular due to the varied nature of the fuel handling systems and the move away from conventional electrolytes. This leads to them being better defined by the state/type of the fuel rather than electrolyte chemistry as this is more relevant to the system design and the end use application of the system. Examples of this are direct methanol or ethanol or carbon fuel cells. This classification system is not ideal as there is significant ambiguity as to in which class a fuel cell should reside. In particular, depending on the operating temperature or pressure, the fuel may be either a gas or a liquid. Figure 7 shows a broad fuel-based classification of different fuel cells currently being investigated and is color coded to give an indication of the potential end user applications for each fuel cell type.

Systems based on solid fuels have the attraction that these fuels are often low cost and more abundant than liquid or gaseous fuels. The gaseous fuels have the advantage of being reasonably abundant and can be easily transported over long distances through conventional pipe networks. Liquid fuels are the least abundant of all of the potential fuel sources but are easy to transport and high energy densities make them most suited to transport or mobile applications.

Within the solid fuel class, there are two fuel cell types that could potentially result in a paradigm shift with respect to power generation and application potential: Microbial Fuel Cells (MFC) and Direct Carbon Fuel Cells (DCFC).

**Figure 8:**
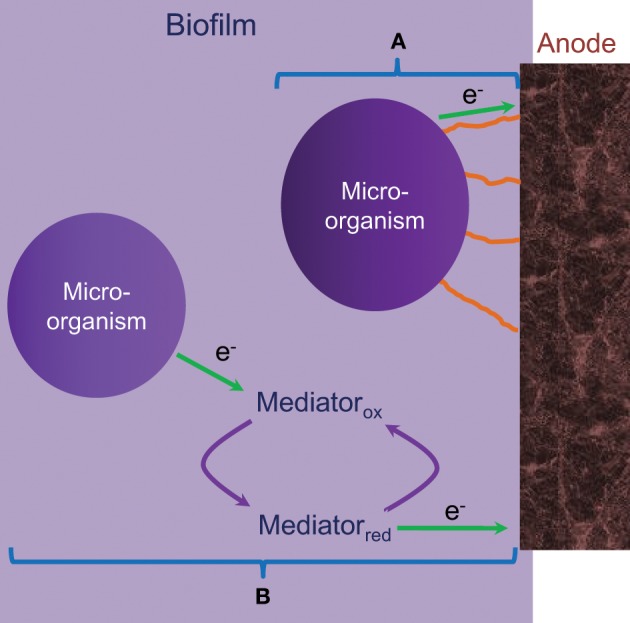
**Two modes of operation of a MFC**. **(A)** Direct reaction, and **(B)** indirect reaction. Figure reproduced from data in Knight et al. (2013).

***Microbial fuel cells (MFC)***. MFC convert organic material into electrical energy via the microbes' metabolic processes. The use of microbes to produce electric current has been explored since the 1970s but has only recently been revisited for use as a power source for small scale applications as higher power densities are being demonstrated (Rabaey et al., 2009). MFC generally take two forms, membrane reactors and single chamber fuel cells. Within a membrane reactor device, the anode and cathode are separated into two chambers by an electrolyte membrane whereas with single chamber devices both the anode and cathode are in one chamber but separated by organic material. The second class are typically referred to as sediment cells. In both classes of MFC, microorganisms form a biofilm on the surface of the anode and oxidize organic material. These microorganisms then transfer electrons to the anode of the fuel cell either directly (Figure 8A) via micro-pili or indirectly via a mediator (Figure 8B).

MFC are considered promising as they operate at or near room temperature and can utilize low grade waste materials such as soils and sediments, waste water and agricultural waste streams that are unsuitable for use in any other power generation technology. The main issue, however, is the very low power density of this type of fuel cells which is typically in the μWcm^−2^ range which is several orders of magnitude below that of other fuel cell types (Rabaey et al., 2009; Knight et al., 2013). Although these fuel cells offer promise in certain low power demand applications, if they are to be adopted at a large scale for such applications, then the power densities need to be increased substantially to at least in the mWcm^−2^ range.

In addition to the absolute performance of MFC's other critical challenges that need to be overcome include faster response times to varying loads, increased voltage stability, increased lifetime, and improved methods of fuel supply to the electrodes. Unlike the majority of other fuel cell types these issues are not fundamentally materials related with the greatest drivers for improvement being novel designs that allow greater mixing of oxidant or fuel with the microbe laden electrodes, improved coupling between the microbes and the electrodes, and selection or modification of the microbes to increase reaction rates at the electrodes. If the activity of the electrodes could be enhanced then further improvements could be obtained via the modifying of the cell design and materials to reduce resistive losses in the electrolyte and electrodes. This could be most easily achieved by reducing the electrolyte thickness or improving the conductivity of the electrodes and/or electrolyte. These improvements are unlike to have a dramatic effect on the performance of MFC's until the activity of the electrodes is increased which would result in higher current passage through the cell. Rabaey et al. (2009) provides a comprehensive technical overview of MFC technology, detailed information regarding the latest understanding of the mechanisms occurring at the electrodes and information on the various designs that are being trialed globally. Carrette et al. (2005) and Knight et al. (2013) provide more generic information on fuel cells with reference Knight et al. (2013) focussing on some of the design strategies that can be used with respect to utilizing MFC's as practical power generation systems.

***Direct carbon fuel cells (DCFC)***. Direct carbon fuel cells and fuel cells that directly electrochemically consume hydrocarbon fuels offer many advantages and could potentially compete in many common market sectors to other fuel cell types. The attraction of direct electrochemical oxidation of carbon or gaseous hydrocarbon fuels is that there is the potential to significantly enhance the electrical efficiency of a fuel cell system if the fuel is directly electrochemically reacted rather than gasified or reformed (Table 1).

The DCFC technology has been described in a considerable detail in a recent review article (Giddey et al., 2012). Some of the benefits of the technology, in addition to high efficiency (>65–70% electric, 90% combined heat and electric), include low CO_2_ emission, and as the by-product of carbon oxidation is CO_2_, its capture costs and energy requirements are very low. Furthermore, if a solid fuel is used (carbon or a high carbon containing hydrocarbon fuel such as coal or biomass chars) then the stability of the fuel becomes less of an issue. These fuels have far higher stability than liquid or gaseous fuel and hence can be fed to the anode surface where they remain stable until oxidized in a chemical or electrochemical reaction.

The DCFC technology is at an early stage of development with a number of different types of DCFC under consideration with a number of groups globally now reporting operation of small stacks (Giddey et al., 2012). Although the electric efficiency is high (>65–70%), reported power densities for these systems, especially once scaled up, are still significantly lower than that of conventional fuel cells operated on gaseous fuels. This is largely due to the reduced surface area for reaction between the anode and the solid fuel that is incapable of infiltrating a porous anode. In order to improve performance a number of groups globally have trialed various strategies to increase the available surface area for reaction. This has included the use of molten metal anodes, molten salt electrolytes, or mixed ionic/electron conducting anode materials (Damian and Irvine, 2012; Giddey et al., 2012; Kulkarni et al., 2012; Jayakumar et al., 2013). A number of these system designs are now in the process of being scaled up with technical issues such as system life, fuel quality, fuel feed, and system cost all still remaining as critical that need to be resolved before these devices can be demonstrated at any significant scale.

As with conventional HT fuel cell systems, the majority of issues currently hindering development of DCFC relate to materials and in particular the way in which materials react with the fuel and other cell components at HTs. In addition to materials issues, there are likely to be an increasing number of challenges relating to fuel handling and processing as this technology matures leading to larger systems being tested for longer periods. Due to the relative immaturity of the field these issues are, as yet, poorly defined.

Dependent on cell design and construction materials issues vary significantly (Giddey et al., 2012). In general reactivity issues are greatest with cell designs that contain molten components in particular molten salts. In cell designs that do not contain solid ion conducting layers, these issues are common with other molten salt fuel cell designs, such as molten carbonate fuel cells, and are relate to the mobility of the electrolyte and its reactivity with other system components (Kulkarni and Giddey, 2013). Although the degradation mechanisms are common with molten carbonate fuel cell designs, the higher operating temperature of DCFC's (typically 800°C vs. 650°C) leads to accelerated degradation rates (Giddey et al., 2012; Kulkarni and Giddey, 2013). The molten salt within the fuel cell can be contained and separated with a dense oxide ion conducting membrane, in this instance the fuel is normally mixed with the molten salt and contained within the anode chamber. This greatly simplifies the issues relating to mobility of the molten components within the fuel cell and results in high power densities but the dense oxide membrane can be rapidly corroded by the molten salt/fuel mixture. Some progress has been made in reducing the reaction rate but this is still seen as a critical issue (Damian and Irvine, 2012; Giddey et al., 2012). A molten metal can operate well as an anode material when a solid fuel is used, however, these metals are likely to be highly reactive toward impurities within the fuels which will accumulate in the anode chamber and result in solidification of the molten metal. These fuel cells are also limited in terms of operating voltage by the reduction potential of the molten metal which can lead to a significant reduction in overall system efficiency (Giddey et al., 2012; Jayakumar et al., 2013). A fuel cell design, where a mixed ionic electronic conducting (MIEC) anode is used to shift carbon oxidation reaction from electrode/electrolyte interface to anode/fuel interface, is likely to have the least reactivity issues due to the fact that all fuel cell components are solid state. This makes them less reactive toward fuel impurities and in general more stable. However, these materials have lower ionic conductivity than molten salts and lower electrical conductivities than molten metals leading to MIEC DCFC's having, in general, lower power densities when compared to other DCFC designs. If stable materials with high mixed ionic and electronic conductivities can be identified, this fuel cell system would rapidly evolve as a leading contender as it can utilize many of the materials and design features of the more technologically mature SOFC technology (Giddey et al., 2012; Kulkarni et al., 2012; Badwal et al., 2014).

#### Small and portable fuel cells

In addition to next generation fuel cell systems that operate on gaseous and solid fuels at ultrahigh efficiencies, there is also a drive to develop small scale or portable power sources. In these systems, device volume and weight, fuel energy density, and ease of transport of the fuel are critical with the overall system efficiency being important but less critical than for stationary power generation. Portable fuel cell systems are generally based on LT PEM fuel cell stacks that operate near room temperature on pure hydrogen with a limited number of systems being developed that are based on either SOFC technology or that are based on PEM systems but that operate directly on methanol/water mixes (Giddey et al., 2012; Badwal et al., 2014). If the fuel cell is to be operated on pure hydrogen then this is normally stored either within a metal hydride or light weight compressed hydrogen cylinder. Other fuels under consideration include bio-fuels such as ethanol, synthetic hydrocarbon fuels such as methanol and non-hydrocarbon fuels such as ammonia (Brown, 2001; Choudhary et al., 2001; Giddey et al., 2013). For fuel cells operated on the non-hydrogen fuels, with the exception of direct methanol fuel cells, a fuel processor is required to convert the fuel into either pure H_2_ or a mixture of H_2_ and CO with the later only being suitable for use in HT fuel cell systems.

The use of a fuel processor can often greatly increase the complexity of the device but simplifies the storage of the fuel, particularly in the case of liquid fuels which can often have exceptionally high energy densities and low cost in comparison to either batteries or gaseous hydrogen storage solutions. However, due to the stringent requirements relating to the purity of hydrogen, the cost of the fuel processor can often significantly increase the overall cost of the device with the fuel processer potentially being greater than the cost of the fuel cells stack itself. This typically limits fuel cell/fuel processor combinations to applications where the cost per kWh is more critical than the cost per kW as this allows the high cost of the fuel processor to be offset by the much reduced cost of the fuel storage solution. Similarly, any additional weight from the processor can be offset by the far higher energy density of the fuel storage solution.

These small and portable fuel cell systems are being developed for a range of end-user applications including stationary backup generators, battery charging, remote area power, auxiliary power units, soldier packs, portable electronic appliances, and small transport applications. There are an increasing number of these devices now commercially available, however, lack of fuel infrastructure and high cost when compared to battery or battery generator combinations remain key challenges that need to be overcome for this market to expand further. Future fuel cell designs should be able to operate directly on a greater variety of commonly available fuels without the requirement for significant amounts of fuel pre-processing. This should lead to far greater efficiencies and hence lower operating costs of fuel cell power systems when compared to conventional power generating technologies which are likely to remain lower cost in terms of capital investment in the medium to long term.

### Alkali Metal Thermo-electrochemical energy converters (AMTEC)

The Alkali-Metal Thermo-electrochemical Converter (AMTEC) is an electrochemical device which utilizes heat from a solar or a nuclear source or from combustion of fossil fuels to generate electricity and is an excellent technology for conversion of heat to electricity (Weber, 1974; Cole, 1983; Ryan, 1999; Lodhi and Daloglu, 2001; El-Genk and Tournier, 2004; Wu et al., 2009). The AMTEC is thermodynamically somewhat similar to the Rankine cycle with conversion efficiencies in the 20–40% range, similar to the Carnot cycle. AMTEC devices offer high efficiency for the operating temperature regime and part-load operation independent of size and high power densities around 1 W/cm^2^. Some applications of AMTEC devices include dispersed small scale power generation, remote power supplies, aerospace power systems, and vehicle propulsion.

**Figure 9:**
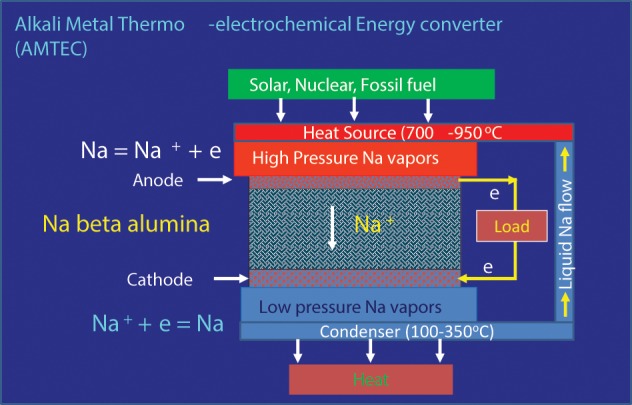
**The operating principle of an Alkali Metal Thermo-electrochemical Energy converter (AMTEC)**.

Typically an AMTEC device consists of a sodium or potassium beta alumina as the electrolyte for the transport of Na^+^ or K^+^ ions and sodium or potassium metal as the fluid that drives the device. These materials are known to have high ionic conductivity with ionic transport number for Na^+^ or K^+^ close to unity (Badwal, 1994). The electrolyte separates the high pressure (>20 kPa) and HT (700–950°C) section of the device from the low pressure (~100 Pa), LT (100–350°C) side of the cell (Weber, 1974; Cole, 1983; Ryan, 1999; Lodhi and Daloglu, 2001; El-Genk and Tournier, 2004; Wu et al., 2009). A schematic of the AMTEC is described in Figure 9 for a system based on sodium as the working fluid. The liquid metal is supplied to one side of the solid electrolyte. With heat provided from an external source, the liquid metal is evaporated and typically Na vapors are present at the porous anode/dense electrolyte interface at a high pressure (>20 kPa). At the cathode, the Na vapor pressure is reasonably low (~100 Pa).

Due to differential partial pressure of Na across the Na^+^ conducting electrolyte membrane, a voltage signal develops (typically in the V range). When the cell is connected through an external load, under this potential gradient, the sodium in the vapor form is ionized to form Na^+^ ions and electrons at the anode/electrolyte interface. The Na^+^ ions are transported across the electrolyte membrane and recombine with electrons at the cathode (low pressure side) thus producing electricity. The sodium vapors are condensed and cycled back to the anode side for revaporization and the cycle is repeated. A number of cells are connected in series/parallel arrangement to construct a module to meet power requirements of an application. There are no moving parts within the cell and therefore the device has low maintenance requirements. The AMTECs are modular in construction and in many respects have common features with batteries and fuel cells.

The technology has been under development since late 1960s with initial effort going into liquid sodium anode based devices. However, due to low cell voltage and power density, more recent effort has been directed toward vapor phase anode or vapor fed liquid anode systems with significant advances made in the development and manufacturing with performance of multi tube modules demonstrated for several thousand hours of operation (Wu et al., 2009). AMTEC systems in the 10s of kW range have been developed and deployed for space applications (Weber, 1974; Cole, 1983; El-Genk and Tournier, 2004; Wu et al., 2009).

Despite the simple operating principle of the AMTEC device and demonstration of the technology at multi kW level, the technology is quite complex with several severe issues still contributing to the cost, system efficiency, and lifetime. These include: stability of electrodes, electrolyte, and other materials of construction during operation leading to cell power degradation with time; sodium fluid flow management including heat removal during condensation on the cathode side to heat input on the anode side; power controls; system design; and low cost technology up-scaling. The electrode materials play a critical role for charge exchange at the electrode–electrolyte interface and contribute significantly to cell performance (efficiency and degradation). A number of different materials ranging from metals to ceramics or composites of metals and ceramics have been tried with varying degrees of success (Wu et al., 2009). The electrolyte material is also prone to changes in electrical, chemical, and thermo-mechanical properties with extended operation leading to degradation with time. Thus, although the technology offers many advantages for an extensive range of applications, further improvements to lifetime, reliability, power density, and efficiency are required.

## Energy storage

The implementation of energy storage for applications including transportation and grid storage has strong commercial prospects. A number of market and technical studies anticipate a growth in global energy storage (Yang et al., 2011; Akhil et al., 2013). The main forecasted growth of energy storage technologies is primarily due to the reduction in the cost of renewable energy generation and issues with grid stability, load leveling, and the high cost of supplying peak load. Additionally, the demand for energy storage technologies such as rechargeable batteries for transportation has also added to the forecasted growth. A number of battery technologies have been commercialized and additionally a large number are still under development.

### Rechargeable metal-air batteries

The development of nearly all electrically powered devices has closely followed that of the batteries that power them. By way of example, the size and form of today's mobile phones is largely determined by the dimensions of the lithium-ion cells that have the required capacity. Electric vehicles for passenger transportation are an obvious exception. Here, the batteries and electric drive are replacing systems based on liquid-fuel fed combustion engines that provide levels of performance (acceleration, distance between refueling, etc.) which are taken for granted by the motoring public. There is general reluctance by vehicle owners to embrace electric cars offering considerably less all-round performance. This is the main factor that drives researchers to look well-beyond current lithium-ion technology to a range of new metal-air batteries. By virtue of removing much of the mass of the positive electrode, metal-air batteries offer the best prospects for achieving specific energy that is comparable with petroleum fuels.

#### Lithium-air (oxygen)

**Figure 10:**
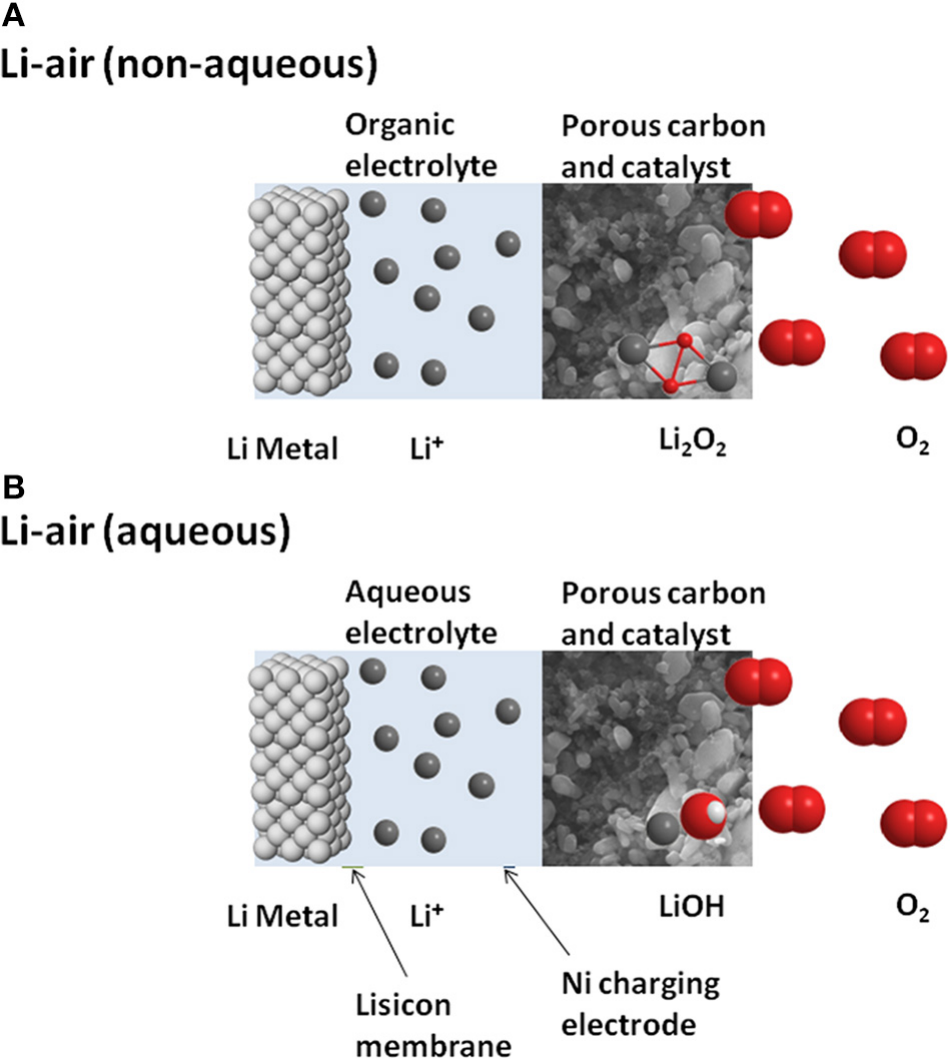
**Schematic representation of two contemporary versions of the lithium-air battery—(A): non-aqueous version, similar to Li-ion; (B): aqueous, with Li^+^-permeable membrane protecting the lithium anode**.

In its simplest form, the lithium-air cell brings together a reversible lithium metal electrode and an oxygen electrode at which a stable oxide species is formed. There are two variants of rechargeable Li-air technology—a non-aqueous and an aqueous form, both of which offer at least ten times the energy-storing capability of the present lithium-ion batteries (Girishkumar et al., 2010; Bruce et al., 2011; Kraytsberg and Ein-Eli, 2011; Imanishi and Yamamoto, 2014). Figure 10 provides a schematic view of the two versions. In both, the cathode is a porous conductive carbon which acts as the substrate for the reduction of oxygen, while the anode is metallic lithium. For the non-aqueous system, the reduction of oxygen ends with formation of peroxide, so that the overall reaction follows Equation (1).

(1) $$2\text{Li}(\text{s})+{\text{O}}_{2}(\text{g})={\text{Li}}_{2}{\text{O}}_{2}(\text{s})$$

A cell based on this reaction has an open circuit voltage of 2.96 V and operates at specific energy values ranging between 3460 and 11,680 Wh kg^−1^. During discharging, the cell draws in oxygen and thereby gains mass, while it loses mass during charging, so that specific energy reaches a maximum when fully charged.

In the aqueous form of lithium-air battery, water is involved in the reduction of oxygen, while the lithium electrode must be protected from reaction with water, usually by means of a lithium-ion-conducting solid electrolyte such as LISICON. Typically the electrolyte solution is a saturated solution of LiCl and LiOH and the favored reduction product is a hydrated lithium hydroxide, according to Equation (2).

(2) $$4\text{Li}(\text{s})+6{\text{H}}_{2}\text{O}(\text{l})+{\text{O}}_{2}(\text{g})=4(\text{LiOH}.{\text{H}}_{2}\text{O})(\text{s})$$

The involvement of water in the reaction complicates the operation of the cell and degrades the specific energy which is theoretically around 2000 Wh kg^−1^ and varies over ~100 Wh kg^−1^ with state-of-charge (Imanishi and Yamamoto, 2014). While this is still an impressive level of performance, the main problem with the aqueous form of lithium-air is the difficulty of maintaining separation of lithium metal from the aqueous medium. Most of the Li^+^-conducting solids tried to date do not have sufficient long-term stability against aqueous solutions. In addition they contribute significantly to cell impedance—reducing the thickness of this protective layer ameliorates this effect but is limited by the poor mechanical strength of very thin layers. For these reasons, most research effort in lithium-air batteries is focusing on the non-aqueous form.

Clearly a key aspect to the realization of the very high specific energy of lithium-air battery is that the lithium metal anode can be made to operate safely and at full utilization. Many early studies used the organic carbonate electrolytes from lithium-ion battery technology, until it was eventually discovered that these compounds (ethylene carbonate, propylene carbonate, etc.) were being oxidized during the charging phase, with the liberation of carbon monoxide and carbon dioxide. Solvents with ether functionality have since taken precedence given that they are more stable during charging and also less likely to promote the growth of dendritic morphologies at the lithium electrode (Abraham and Jiang, 1996). Nevertheless, both carbonates and ethers are flammable which ultimately makes these devices hazardous under conditions where they become hot. It is not surprising therefore that interest has turned to the use of ionic liquids, which are essentially non-volatile and able to dissolve appreciable concentrations of most lithium salts. In addition, lithium electrodes operate with a high degree of reversibility in a range of low viscosity ionic liquid media, without the formation of dendrites, due to the formation of a durable solid electrolyte interphase (SEI) on lithium (Howlett et al., 2004). An increasingly attractive option to the metallic lithium electrode is to use one of the high capacity lithium host materials, notably silicon which offers the prospect of almost 2000 mAh g^−1^ by accessing the full available storage limit (based on Li_4.4_Si).

The positive electrode of a lithium-air cell represents a complex challenge in that it must provide for: (i) access to oxygen; (ii) wetting by the electrolyte; and (iii) displacement by reaction products. While allowing access to oxygen, the electrode must be able to block access to water, carbon dioxide, and nitrogen, which will all react with the electrode materials and/or products of reaction at the electrodes. The properties of the main product of discharge, lithium peroxide, Li_2_O_2_, also pose a number of problems with regard to cell longevity. First, it is an insulating solid, which means that conditions must be adjusted to prevent the formation of massive deposits during discharging. Second, lithium peroxide is a strong oxidant that tends to react with electrolyte components, including any adventitious water, to form irreversibly a variety of materials that severely degrade the lifetime of a Li-air cell.

In the last few years, researchers have been able to extract something close to the high levels of performance that the lithium-air system offers, but only for brief periods before rapid capacity loss occurs. The reversibility of oxygen reduction is still the key issue (Mo et al., 2011), and even when conditions are adjusted to promote chemical reversibility, there is a large overvoltage associated with charging which will ultimately work against developing fast-charging procedures. Accordingly, there is still considerable investigation required into the exact mechanism of oxygen reduction, and the oxidation of a range of oxide species, with the aim of greatly improving the energetics of these processes.

#### Sodium-air (oxygen)

The reversible sodium electrode is well-known in the history of battery development as it is featured in some of the very earliest examples of high performance secondary batteries. Both the sodium-sulfur and the Zebra (sodium-nickel chloride) systems employ molten sodium electrodes which give reversible behavior at values of potential that are sufficiently negative for useful device voltages (Ellis and Nazar, 2012). Recently, the sodium electrode has again become the focus of attention, now coupled with an oxygen electrode in the sodium-air cell. This, like all metal-air systems, benefits in energy terms from the inherently lightweight air-breathing cathode and offers theoretical values of specific energy that range from 1105 to 2643 Wh kg^−1^, depending on the state-of-charge. These numbers are derived from the overall cell reaction shown in Equation (3).

(3) $$\text{Na}(\text{s})+{\text{O}}_{2}(\text{g})={\text{NaO}}_{2}(\text{s})$$

The identification of the superoxide as the main product of reduction has been verified experimentally (Hartmann et al., 2013) and although a basic thermodynamic treatment indicates that it is not the favored product, Ceder's Group has shown that when the discharge products are nanostructured, the surface energetics make the superoxide the preferred product phase (Kang et al., 2014).

Many of the limitations on performance of the air cathode in Li-O_2_ cells also define the behavior of this electrode in Na-O_2_ cells. The use of carbonate and ether electrolyte solutions has been hampered by problems of insufficient stability during charging (Hartmann et al., 2013). While the preferential formation of sodium superoxide during discharging clearly lowers the overpotential associated with charging, it is not clear whether this compound will be stable on the longer timescale of a typical device service life, or whether the discharge product will gradually be converted to the more stable, and less easily recharged, sodium peroxide.

While the molten sodium electrode offers many advantages in terms of electrochemical characteristics, reality for rechargeable energy storage devices demands that maximum performance is delivered at ambient temperature. What is known of the behavior of solid sodium electrodes in conventional battery electrolytes suggests that it readily generates dendritic morphologies thereby posing a significant risk to further development of this battery technology. By analogy with lithium electrochemistry, it seems likely that more attention will be given to examining the behavior of sodium in ionic liquid electrolytes, in an attempt to replicate the benefits of generating a protective SEI in a medium that is inherently safer with respect to volatility and reactivity.

Although it is very early in the development cycle for sodium-air batteries, there are sound reasons for pursuing further progress. The relative abundance of sodium, compared with lithium, is perhaps the most obvious, and the fact that sodium is close to lithium in terms of mass and electrochemical potential also strengthen the case. Continued larger efforts to develop positive electrode substrates for other metal-air systems (notably lithium) will directly benefit the sodium-air positive electrode. With research already appearing on non-volatile sodium ion-conducting electrolytes based on ionic liquids, it would seem that the main issues holding back the development of sodium-air batteries are now being addressed.

### Lithium-sulfur batteries

**Figure 11:**
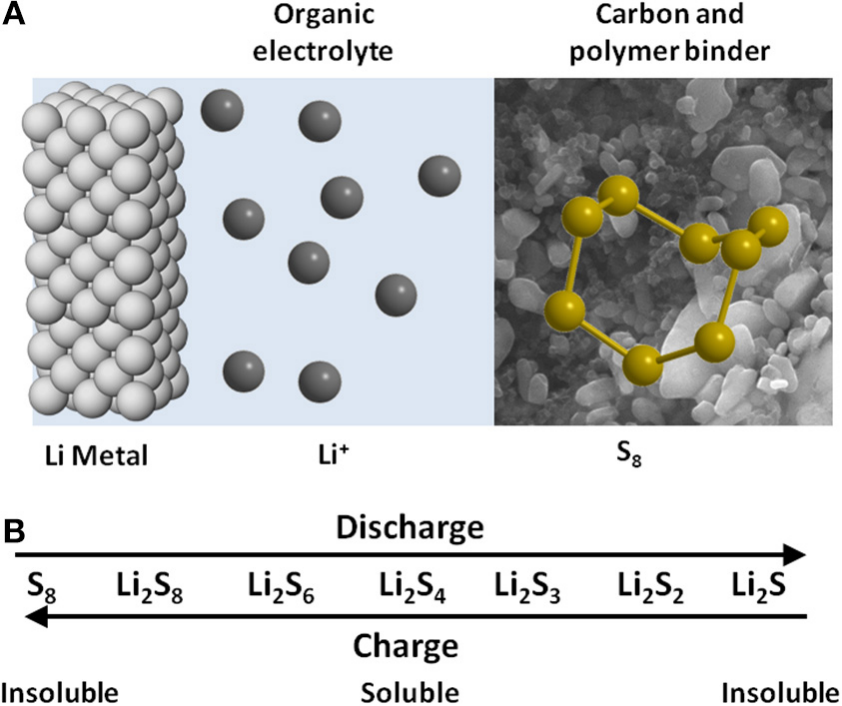
**(A)** A schematic view of the lithium-sulfur cell. **(B)** Summary of reactions that define Li-S and their relationship with solubility.

A positive electrode comprised solely of elemental sulfur has a theoretical specific capacity of 1672 mAh g^−1^. Assuming an equivalent amount of lithium for the negative electrode, complete reaction of Li and S to form Li_2_S, and an average discharge potential of 2.2 V per cell, the electrode specific energy for Li-S is 2600 Wh kg^−1^ (Bruce et al., 2012; Manthiram and Su, 2013; Song et al., 2013). The overall discharge reaction, in its simplest form, is given in Equation (4), and a schematic view of the components and their role is provided in Figure 11.

(4) $$2\text{Li}(\text{s})+\text{S}(\text{s})\to {\text{Li}}_{2}\text{S}(\text{s})$$

Fully packaged, it is expected that Li-S batteries in real life will operate at up to 700 Wh kg^−1^. This level of performance places lithium-sulfur well-clear of existing battery systems, and many view it as a logical intermediate step to the lithium-air battery. In many ways, lithium-sulfur also poses a set of mid-level challenges to battery researchers.

While not sharing the full range of difficulties of the air electrode, the sulfur electrode still represents a complex electrochemical system in which elemental sulfur, in the form of S_8_ molecules, is successively reduced through a sequence of polysulfide dianions (Bruce et al., 2012). The solubility of the lithium salt of each successive reduction product decreases appreciably, with the end discharge product, Li_2_S, being virtually insoluble in common organic electrolyte media. Overlaying this is the generally labile nature of exchange between intermediate members of the polysulfide series, which has the undesirable consequence of allowing significant loss of efficiency through a redox shuttle phenomenon (Manthiram and Su, 2013). As a result of these solution-based issues, most research groups strive to minimize the solubility of polysulfides in the electrolyte.

As it happens, however, controlling the solubility of sulfur and its reduction products is not sufficient on its own to stabilize the performance of the lithium-sulfur battery. It is now clear that the positive electrode, which is the mechanical support for sulfur, must not only be conductive, but also mesoporous, to maximize electrode area within dimensions that do not restrict ion diffusion, and to incorporate surface functionality that acts to adsorb polysulfides so as to enhance the retention of discharge products within the positive electrode. With this knowledge, the design of sulfur positive electrodes now typically incorporates additives such as mesoporous silica, to enhance retention of polysulfides within the electrode, and nano-structured polymer films with chemical functionality to restrict the flow of sulfur species out of the electrode.

In the presence of sulfur and polysulfides, the use of lithium metal as the negative electrode is more complicated than in other lithium battery systems due to a range of interactions between metallic lithium, sulfur species, and electrode-stabilizing additives such as lithium nitrate (Aurbach et al., 2009). Helping to provide greater control over the behavior of the lithium electrode is the increasing trend to incorporate ionic liquids in Li-S electrolyte blends. Here it is the fluorosulfonyl imide anions (either FSI or TFSI), which contribute to the formation of a stable SEI, that provide the basis for safe, dendrite-free operation of the lithium negative electrode. More recently, it has also been discovered that lithium ion transport characteristics can be greatly enhanced, while at the same time suppressing the solubility of polysulfide species, by increasing the concentration of the lithium salt to unprecedented levels (>5 M).

Despite the high degree of chemical complexity inherent to the lithium-sulfur battery, there are strong signs that the issues which have thwarted progress are now being brought under control, mainly through the tailoring of electrode and electrolyte materials to deal with specific aspects of performance. At the same time, it is interesting to note that the development of lithium-sulfur battery technology also seems likely to give rise to a successful all-solid component version, due to the advent of a family of high-lithium-ion-conducting ceramic sulfides (Kamaya et al., 2011).

### Flow batteries

A flow battery is a rechargeable battery where the energy is stored in one or more electroactive species dissolved into liquid electrolytes. The electrolytes are stored externally in tanks and pumped through electrochemical cells which convert chemical energy directly to electricity and vice versa, on demand. The power density is defined by the size and design of the electrochemical cell whereas the energy density or output depends on the size of tanks. With this characteristic, flow batteries can be fitted to a wide range of stationary applications. Originally developed by NASA in the early 1970's as electrochemical energy storage systems for long-term space flights, flow batteries are now receiving attention for storing energy for durations of hours or days. Flow batteries are classified into Redox flow batteries and hybrid flow batteries.

Flow batteries have the advantages of low cost devices, modularity, easy transportability, high efficiency and can be deployed at a large scale (Ponce de Leon et al., 2006). The modularity and scalability of these devices means they can easily span the kW to MW range. As a result, their main development at present is focussed on standalone remote area power systems or grid energy storage/support in combination with renewable energy generation (Skyllas-Kazacos et al., 2011).

#### Redox flow battery (RFB)

In redox flow batteries (RFB), two liquid electrolytes containing dissolved metal ions as active masses are pumped to the opposite sides of the electrochemical cell. The electrolytes at the negative and positive electrodes are called negative electrolyte (also referred to as the anolyte) and positive electrolyte (also referred to as the catholyte), respectively. During charging and discharging the metal ions stay dissolved in the fluid electrolyte; no phase change of these active masses takes place. Negative and positive electrolytes flow through porous electrodes, separated by a membrane which allows protons to pass through it for the electron transfer process. During the exchange of charge a current flows over the electrodes, which can be used by a battery-powered device. During discharge the electrodes are continually supplied with the dissolved active masses from the tanks; once they are converted, the resulting product is removed to the tank.

Various redox couples have been investigated and tested in RFBs, such as a Fe-Ti system, a Fe-Cr system, and a polyS-Br system. The vanadium redox flow battery (VRFB) has been developed the furthest; it has been piloted since around 2000 by companies such as Prudent Energy (CN) and Cellstrom (AU). The VRFB uses a V^2+^/V^3+^ redox couple as the negative pair and a V^5+^/V^4+^ redox couple in mild sulfuric acid solution as the positive pair. The main advantage of this battery is the use of ions of the same metal on both sides. Although crossing of metal ions over the membrane cannot be prevented completely (as is the case for every Redox flow battery), in VRFBs the only result is a small loss in energy. In other RFBs, which use ions of different metals, the crossover causes an irreversible degradation of the electrolytes and a loss in capacity. The VRFB was pioneered at the University of New South Wales, Australia, in the early 1980s (Skyllas-Kazacos et al., 2011).

#### Hybrid flow battery (HFB)

In a hybrid flow battery (HFB) one of the active masses is internally stored within the electrochemical cell, whereas the other remains in the liquid electrolyte and is stored externally in a tank. Therefore, hybrid flow cells combine features of conventional secondary batteries and redox flow batteries: the capacity of the battery depends on the size of the electrochemical cell. Typical examples of a HFB are the Zn-Ce (Fang et al., 2002; Clarke et al., 2004; Ponce de Leon et al., 2006; Reddy, 2011) and more commonly the Zn-Br_2_ system (Lim et al., 1977; Lex and Jonshagen, 1999; Ponce de Leon et al., 2006; Reddy, 2011). In both cases the negative electrolyte consists of an acid solution of Zn^2+^ ions. During charging Zn is deposited at the electrode and on discharging Zn^2+^ goes back into solution. In the case of the Zn-Br systems the electrode reactions are shown below:

During discharge, the zinc in the anode is oxidized:

(5) $$\text{Zn }\overset{\text{discharge}}{\underset{\text{charge}}{⇄}}\;{\text{Zn}}^{2+}+2\text{e }-0.763\text{V}$$

At the cathode bromine is reduced, to bromide, Br^−^,

(6) $${\text{Br}}_{2}+2\text{e }\overset{\text{discharge}}{\underset{\text{charge}}{⇄}}\;2{\text{Br}}^{-}\;+1.087\text{V }$$

so that the overall reaction is:

(7) $$\text{Zn}+{\text{Br}}_{2}\;\overset{\text{discharge}}{\underset{\text{charge}}{⇄}}\;{\text{Zn}}^{2+}+2{\text{Br}}^{-}\;+1.850\text{V}.$$

The two electrode chambers of each cell are separated by a membrane (typically a microporous or ion-exchange variety). This helps to prevent bromine from reaching the positive electrode, where it would react with zinc, causing the battery to self-discharge. To further reduce self-discharge and to reduce the vapor pressure of bromine, complexing agents are added to the positive electrolyte. These react reversibly with the bromine to form an aqueous solution and reduce the free Br_2_ in the electrolyte. The working electrodes in the Zn-Br_2_ battery are based on carbon-plastic composites.

Various companies are working on the commercialization of the Zn-Br_2_ hybrid flow battery, which was developed by Exxon in the early 1970s. In the United States, ZBB Energy and Premium Power sell trailer-transportable Zn-Br_2_ systems with unit capacities of up to 1 mW/3 mWh for utility-scale applications. Some 5 kW/20 kWh systems for community energy storage are in development as well. In Australia, Redflow Ltd. has developed a Zn-Br_2_ system for electrical energy storage applications. Zn-Br_2_ batteries can be 100% discharged every day without being damaged and this can be repeated for over 2000 cycles.

#### Flow battery future prospects

In addition to the V- and Br_2_-based systems, a number of alternative chemistries are also being investigated. The reason for this is that the new applications for these devices, such as electricity grid integration, require that the performance, in particular the volumetric energy density is increased. There are a number of challenges still to be overcome to achieve this goal. Firstly, electrode development should focus on porous and catalytic electrodes which allow high electrolyte linear flow velocities to enhance rate capability (Ponce de Leon et al., 2006). Secondly, the engineering of the device also requires attention in the areas of reactor design, electrode materials to enhance catalysis (Ponce de Leon et al., 2006), membrane performance (Ponce de Leon et al., 2006) to reduce migration of active species and finally the large scale engineering (Ponce de Leon et al., 2006) to allow for up-scaling of the technology for very large installations with focus on minimizing maintenance and increasing life. Two very good review articles by Ponce de Leon et al. (2006) and Skyllas-Kazacos et al. (2011) have given a good overview of the development and challenges of flow batteries. Table 2 summarizes the range of different flow battery chemistries which have been previously reported.

**Table 2:** **Summary of flow battery chemistries reported in the recent literature**.

**System**	**Electrode reactions**	**Electrolyte**	**OCP**	**References**
All-vanadium	Negative electrode:	1.6–2 M vanadium sulfate in sulfuric acid in both half-cells	1.6	Skyllas-Kazacos and Grossmith, 1987; Skyllas-Kazacos, 2009; You et al., 2009; Jia et al., 2010; Skyllas-Kazacos et al., 2010
	V^3+^ + e^−^ → V^2+^			
	Positive electrode:			
	VO^2+^ + H_2_O − e^−^ → VO^+^_2_ + 2H^+^			
Vanadium bromine	Positive electrode:	1–3 M vanadium bromide in 7–9 M HBr plus 1.5–2 M HCl in both half-cells	1.4	Skyllas-Kazacos, 2003; Skyllas-Kazacos et al., 2010
	2VBr_3_ + 2e^−^ → 2VBr_2_ + 2Br^−^			
	Negative electrode:			
	2Br− + Cl^−^ → ClBr_2_ + 2e^−^			
Magnesium-vanadium	Positive electrode:	Positive half-cell: 0.3 M Mn(II)/Mn(III) in sulfuric acid)	1.66	Xue et al., 2008
	Mn(II) → Mn(III) + e^−^			
	Negative electrode:	Negative half-cell: V(III)/V(II) in 5 M sulfuric acid		
	V(III) + e^−^ → V(II)			
Vanadium cerium	Positive electrode:	Positive half-cell: 600 ml of 0.5 M Ce(III) in 1 M H_2_SO_4_	1.05	Paulenova et al., 2002; Xia et al., 2002; Leung et al., 2011a
	Ce^3+^ → Ce^4+^ + e^−^			
	Negative electrode:	Negative halfcell: 600 ml of 0.5 MV(III) in 1 M H_2_SO_4_		
	V^3+^ + e^−^ → V^2+^			
Vanadium glyoxal(O_2_)	Positive electrode:	Positive half-cell: 50 ml glyoxal–HCl solution of different concentration	1.2	Wen et al., 2008a
	[OC]_RE_ + H_2_O→ [OC]_OX_ + 2H^+^ + 2e^−^ ([OC]_RE_ = organic reductive materials and [OC]_OX_ = electro-oxidized organic products).			
	Negative Electrode:	Negative half-cell: 1–2 M V(III) + 3 M H_2_SO_4_ solution		
	V^3+^ + e^−^ → V^2+^			
Vanadium cystine (O_2_)	Positive electrode:	Positive half-cell: 0.1 M cystine dissolved in HBr aqueous solution of different concentrations	1.315	Wen et al., 2008b
	RSSR + Br_2_ + 6H2O → 2RSO_3_H + 10HBr (where RSSR = L-cystine and RSO_3_H = L-cysteic acid)			
	Negative electrode:	Negative half-cell: 50 ml of 1 M V(III) + 3 M H_2_SO_4_		
	V^3+^ + e^−^ → V^2+^			
Vanadium polyhalide	Positive electrode:	Positive half-cell: 1 M NaBr in 1.5 M HCl	1.3	Skyllas-Kazacos, 2003
	Br^−^ + 2Cl^−^ → BrCl^−^_2_ + 2e^−^			
	Negative electrode:	Negative half-cell: 1 M VCl_3_ in 1.5 M HCl		
	VCl_3_ + e^−^ → VCl_2_ + Cl^−^			
Vanadium acetylacetonate	Positive electrode:	0.01 M V(acac)_3_/0.5 M	2.2	Liu et al., 2009
	V(III)(acac)_3_ → [V(IV)(acac)_3_]^+^ + e^−^.	TEABF_4_/CH_3_CN in both half-cells		
	Negative electrode:			
	V(III)(acac)_3_ + e^−^→ [V(II)(acac)_3_]^−^			
Vanadium/air system	Positive electrode:	Positive half-cell: H_2_O/O_2_	_1 V for 8 h	Hiroko et al., 1994, 1997
	2H_2_O → 4H^+^ + O_2_ + 4e^−^			
	Negative Electrode:	Negative half-cell: 2 M V^2+^/V^3+^ solution in 3 M H_2_SO_4_		
	V^3+^ + e^−^ → V^2+^			
Bromine polysulfide	Positive electrode:	5 M NaBr saturated with Br_2_ and 1.2 M Na_2_S	1.7–2.1	Remick and Ang, 1984; Zhao et al., 2005; Zhou et al., 2006
	3Br^−^ → Br^−^_3_ + 2e^−^			
	Negative electrode:			
	S^2−^_4_ + 2e^−^ → 2S^2−^_2_			
Zinc-bromine	Positive electrode:	1–7.7 mol dm^−3^ ZnBr_2_ with an excess of Br_2_ with additives such as KCl or NaCl	1.6	Eustace, 1980; Zhou et al., 2006; Nyugen and Savinell, 2010; Weber et al., 2011
	2Br^−^ → Br_2_ + 2e^−^			
	Negative electrode:			
	Zn^2+^ + 2e^−^ → Zn^0(s)^			
Zinc-cerium	Positive electrode:	Anolyte: 0.3 M Ce_2_(CO_3_)_3_ and 1.3 M ZnO in 70 wt.% methanesulfonic acid	2.45	Eustace, 1980; Zhou et al., 2006; Leung et al., 2011a,b
	2Ce^3+^ → 2Ce^4+^ + 2e^−^			
	Negative electrode:	catholyte: 0.36 M Ce_2_(CO_3_)_3_ and 0.9 M ZnO in 995 g methanesulfonic acid		
	Zn^2+^ + 2e^−^ → Zn^0^_(s)_			
Soluble lead-acid	Positive electrode:	Soluble lead (II) species in methanesulfonic acid	1.62	Hazza et al., 2005; Zhou et al., 2006; Collins et al., 2010
	Pb^2+^ + 2H_2_O → PbO_2_ + 2H^+^ + 2e^−^			
	Negative electrode:			
	Pb^2+^ + 2e^−^ → Pb(s)			
All-neptunium	Positive electrode:	1 M nitric acidic solution of 0.05 M neptunium	1.3	Hasegawa et al., 2005; Yamamura et al., 2006a
	Np^3+^ → Np^4+^ + e^−^			
	Negative electrode:			
	NpO^2+^_2_ + 2e− → NpO^+^_2_			
All-uranium	Positive electrode:	U(VI)/U(V)	1.1	Yamamura et al., 2002, 2004, 2006b; Shirasaki et al., 2006a,b
	U(IV) → U(V) + e^−^	β-diketonate solution as the catholyte and U(IV)/U(III)		
	Negative electrode:			
	U(IV) + e^−^ → U(III)	β-diketonate solution as the anolyte		
All-chromium	Positive electrode:	0.2 M chromium EDTA complex in HCl	2.11	Chieng and Skyllas-Kazacos, 1992; Bae, 2001; Bae et al., 2011
	[Cr(III)EDTA(H_2_O)]^−^ → [Cr(V)EDTA(H_2_O)]^+^ +2e^−^			
	Negative electrode:			
	2[Cr(III)EDTA(H_2_O)]^−^ + 2e^−^ → 2[Cr(II)EDTA(H_2_O)]^2−^			
Zinc-air	Positive electrode:	0.4 M ZnO in 6 M KOH solution was employed as the catholyte and propanol of different concentrations in 6 M KOH solution was employed as the anolyte	1.705	Wen et al., 2009
	Propanol oxidation during charging; oxygen reduction during discharge.			
	Negative electrode:			
	Zn(OH)^2−^_4_ +2e^−^ → Zn + 4OH^−^			
Tiron	Positive electrode:	0.25 M Tiron in 3 M H_2_SO_4_ as cathodic active species and the lead electrode as anodic active species	1.10	Xu et al., 2010
	[Tiron] + 2H^+^ +2e^−^ → [Tiron]^−^			
	Negative electrode:			
	Pb + SO^2−^_4_ → PbSO_4_ + 2e^−^			
Zinc-nickel	Positive electrode:	Highly concentrated solutions of ZnO in aqueous KOH	1.705	Cheng et al., 2007; Zhang et al., 2008
	2NiOOH + 2H_2_O + 2e^−^ → 2Ni(OH)_2_ + 2OH^−^			
	Negative electrode:			
	Zn + 4OH^−^ → Zn(OH)^2−^_4_ + 2e^−^			
[Ru(acac)3]	Positive electrode:	0.02 M ruthenium acetylacetonate with 0.1 M tetraethylammonium tetrafluoroborate dissolved in acetonitrile	1.76	Sum and Skyllas-Kazacos, 1985; Chakrabarti et al., 2007
	Ru(acac)_3_] → [Ru(acac)_3_]^+^ + e^−^			
	Negative electrode:			
	[Ru(acac)_3_] + e^−^ → [Ru(acac)_3_]^−^			
Cr(acac)3	Positive electrode:	0.05 M Cr(acac)_3_ and 0.5 M TEABF_4_ dissolved in acetonitrile	3.4	Liu et al., 2010
	Cr(acac)_3_] → [Cr(acac)_3_]^+^ + e^−^			
	Negative electrode:			
	[Cr(acac)_3_] + e^−^ →[Cr(acac)_3_]^−^			
Iron chromium	Positive electrode:	1 M CrCl_3_ and FeCl_2_ in 2 M HCl in the negative and positive sides of the cell, respectively	1.18	Zhou et al., 2006
	Fe^2+^ → Fe^3+^ + e^−^			
	Negative electrode:			
	Cr3^+^ + e^−^ → Cr^2+^			

*Reproduced from data presented by Skyllas-Kazacos et al. (2011) and Ponce de Leon et al. (2006)*.

An emerging concept for flow batteries is the use of microfluidics to remove the membranes from the system. These devices use laminar interfaces between the positive and negative electrolyte streams to separate the reactants. This approach offers the flexibility that allows the exploitation of a much wider range of chemistries. In the literature, chemistries such as vanadium redox flow batteries (Salloum and Posner, 2010, 2011) and a hybrid hydrogen-bromine flow battery (Braff et al., 2013) have been reported. Typically, the devices have power capabilities in the 0.25 W/cm2 [borohydride-cerium ammonium nitrate (Da Mota et al., 2012) to 0.795 (hydrogen-bromine flow cell Braff et al., 2013)]. This approach allows high efficiencies in the 90% range to be obtained (Braff et al., 2013). Although, the prospects for membrane-less flow batteries looks promising, significant work is still left to do before these devices can become a commercial reality.

### Supercapacitors

Supercapacitors are electrochemical devices that store energy by virtue of the separation of charge, unlike batteries, which store energy through chemical transformation of electrode materials. Known also as ultracapacitors, supercapacitors continue to develop and mature as an energy storage technology, though somewhat still in the shadow of rechargeable batteries. While the designations “ultra” and “super” reflect the fact that these devices have much higher levels of capacitance than traditional capacitors (so-called “electrolytic capacitors,” etc.), a more useful, but less popular, name is “electrochemical double-layer” capacitor, which reflects the origins of the very high values of specific capacitance in the electrochemical double-layer that forms at the electrode-electrolyte interface. On this basis, supercapacitors were originally “symmetrical” devices based on two identical electrodes, each comprised of a network of activated carbon particles (Zhang and Zhao, 2009). The latter material provided the very high levels of surface area that are required to give reasonable values of specific energy. This parameter is still the main problem for supercapacitors as, while their specific power (up to several kW kg^−1^ for complete devices) is unrivaled, most electrical storage applications require more than 10 Wh kg^−1^ of specific energy (usually a great deal more) and supercapacitors generally struggle to store more than 5 Wh kg^−1^ (Burke, 2010).

**Figure 12:**
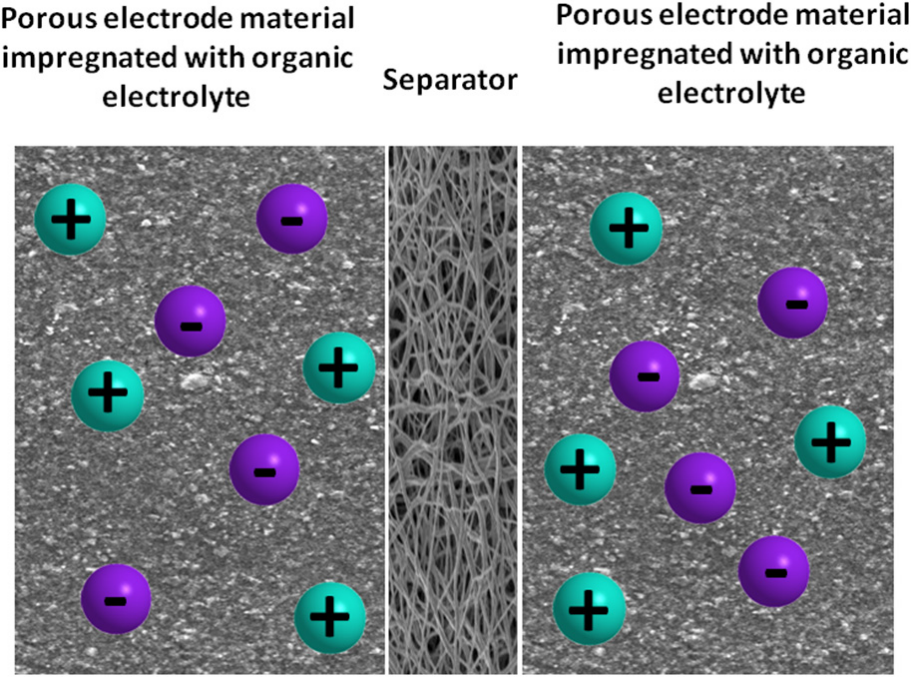
**A schematic view of an electrochemical double-layer capacitor, based on a symmetrical carbon-carbon device**.

Figure 12 summarizes the essential characteristics of a supercapacitor in a schematic form. The electrodes in a carbon symmetrical device are identical, although the respective loading of active materials will be adjusted in line with small variation of specific capacitance for the different ions that make up the supporting electrolyte. In early devices, strong aqueous electrolytes (e.g., sulfuric acid, sodium hydroxide solutions) were employed as the very high values of ionic conductivity led to maximum power outputs. The device voltage was however limited to around 1 V and this has a great impact on specific energy, courtesy of the squared relationship between capacitor voltage and energy. In the last decade, developments have focused on non-aqueous electrolytes with which it has been possible to gradually raise device voltages up to around 2.7 V (Burke, 2010). Given that these electrolyte solutions are based on flammable solvents (acetonitrile, propylene carbonate, etc.) some recent efforts have also been devoted to employing low viscosity ionic liquids in making inherently safer supercapacitors.

Having noted that “traditional” carbon supercapacitors have not progressed beyond 5 Wh kg^−1^ in specific energy, it is not surprising that research in more recent years has turned to ways of moving to much higher values. The phenomenon known as “pseudocapacitance” has been known for many years and was originally detailed in research on the behavior of ruthenium oxide in aqueous media. Materials like this can be repetitively charged and discharged at 5–10 times the specific energy of carbon supercapacitors via a mechanism that involves movement of highly mobile species (hydrated protons or hydroxide ions, depending on pH) to balance changes of charge of the metal oxide active material. Therefore, these devices have a “Faradaic” basis of operation but are not reversible in a Nernstian sense. With chemical change to the electrode materials clearly involved in the mechanism of charge storage, this inevitably leads to internal stresses during charge–discharge which limits cycle-life to values in the 10,000s—well-short of those obtained with symmetrical carbon devices (up to a million cycles). Nevertheless, impressive gains in specific energy have been made with supercapacitors employing manganese oxides (Wei et al., 2011) and conducting polymers (Snook et al., 2011), both of which draw on pseudocapacitance for energy storage.

As a further progression of the ideas to improve specific energy by introducing materials with greater “energy content” per unit weight, a significant stream of research is now developing so-called “hybrid supercapacitors.” These devices incorporate one high surface area carbon electrode and one battery electrode. The latter must be made from a material that is capable of operating at very high rates of charge/discharge, otherwise the performance will not justify the designation as a supercapacitor. To date, the major successes in this field have come with the use of lithium titanium oxide (Li_4_Ti_5_O_12_, LTO) (Naoi et al., 2013). This material works in this role, where others have failed, because it undergoes virtually no dimensional change between charged and discharged states.

Finally, carbon researchers have been far from idle and there have been marked renewals of interest in carbon supercapacitors due to the development of advanced electrode materials based on nanotubes (Fisher et al., 2013) and graphene (Dong et al., 2013). Both forms of carbon are not only highly conductive and therefore excellent bases for capacitor electrodes, but they also provide excellent supports for chemical modifications with which pseudocapacitance can be incorporated. Graphenes in particular have also been shown to be excellent templates for the mesoporous electrode morhpologies that are essential for balancing the dual requirements of conductivity and ion diffusion. There are strong grounds for confidence in the further development of high power devices with enhanced energy storage capability.

### Advanced Pb acid batteries

The lead acid battery is one of the most well-known battery technologies to date first demonstrated by Plante in 1859 (Kurzweil, 2010). The lead acid battery is widely used in a variety of applications including automotive, industrial, submarine, and back-up power amongst many others. The lead acid battery is based on the reactions of lead compounds with sulfuric acid in an electrochemical cell. The discharge reaction equations are as shown below.

At the anode:

(8) $$\text{Pb}+{\text{SO}}_{4}^{2-}\;\overset{{}_{\text{discharge}}}{\underset{{}_{\text{charge}}}{⇄}}\;{\text{PbSO}}_{4}+2{\text{e}}^{-}.$$

At the cathode:

(9) $${\text{PbO}}_{2}+{\text{SO}}_{4}^{2-}+4{\text{H}}^{+}+2{\text{e}}^{-}\;\overset{{}_{\text{discharge}}}{\underset{{}_{\text{charge}}}{⇄}}\;{\text{PbSO}}_{4}+2{\text{H}}_{2}\text{O}$$

so that the overall reaction equation is:

(10) $$\text{Pb}+{\text{PbO}}_{2}+2{\text{H}}_{2}{\text{SO}}_{4}\;\overset{\text{discharge}}{\underset{\text{charge}}{⇄}}\;2{\text{PbSO}}_{4}+2{\text{H}}_{2}\text{O}.$$

There are two different types of lead-acid batteries. The flooded type is the cheapest and tends to be used in automotive and industrial applications. However, the sealed type, also called valve-regulated lead-acid (VRLA), has been rapidly developed and used in a wide range of applications including hybrid and electric vehicles (Cooper, 2004) and power supplies, such as uninterruptible (UPS) and standalone remote areas power supply (RAPS). The sealed/VRLA type, either with absorptive glass mat (AGM) separators or gelled electrolyte technology, has the advantage of low maintenance (due to acid restriction and oxygen recombination) and easy fit configuration. Both the power and energy capacities of lead-acid batteries are based on the size and geometry of the electrodes. The power capacity can be improved by increasing the surface area for each electrode, which means greater quantities of thinner electrode plates in the battery.

Some advantages of the lead-acid system are its low cost, high power, and most successful recycling rate. One disadvantage of lead acid batteries is usable capacity decrease when high power is discharged. For example, if a battery is discharged in 1 h, only about 50–70% of the rated capacity is available. Other drawbacks are lower energy density and the use of lead, a hazardous material prohibited or restricted in various jurisdictions. Advantages are a favorable cost/performance ratio, easy recyclability and a simple charging technology.

It is due to the power performance drawbacks (Yan et al., 2004) that research into advanced hybrid lead acid systems was instigated. Under high-rate partial state-of-charge cycling applications, the lead acid (VRLA type) battery fails prematurely due to the sulfation of the plates (Catherino et al., 2004; Lam et al., 2004). The negative plates suffer from a progressive build-up of lead sulfate which is difficult to remove during recharge. The accumulation of lead sulfate markedly reduces the effective surface-area so that the plate can no longer deliver and accept the required power.

Two approaches exist to overcome this problem. The first is the connection of a supercapacitor device to take up the power requirements and thereby reduce the sulfation issues faced by the plates. However, this option requires sophisticated electronics and control algorithms which results in a complex device to construct. The second approach, taken by Lam et al., was to combine a supercapacitor and a lead-acid battery within the cell, thereby removing the need for control electronics (Lam and Louey, 2006). In this approach the lead acid cell comprises one lead oxide plate and one sponge lead plate. In addition, the negative lead plate also comprises a carbon-based electrode which uses the lead oxide plate as the counter electrode, thereby forming an asymmetric supercapacitor. Thus during operation, the carbon component of the lead/carbon plate acts to buffer high currents from the lead component thereby allowing an increase in power performance and overall battery life. Overall evaluation of the hybrid battery has demonstrated that the technology has a similar working potential to that of the conventional lead acid battery, low hydrogen gassing rates, higher capacity, long cycle life and can easily be manufactured in existing lead acid battery factories. Further evaluation of this technology with new applications such as grid integration with renewable has demonstrated improved performance and greater cycle life than conventional lead acid batteries.

### Storage for renewable generation integration into electricity grids

Currently, significant efforts around the world are placed at reducing CO_2_ emissions in an effort to mitigate climate change issues caused by excess CO_2_ in the atmosphere. In 2011, worldwide 32,600 million tonnes of CO_2_ was emitted from the consumption of energy worldwide (International Energy Statistics, 2011). Of the global energy being produced, over 80% is fossil fuels based (WEO, 2012). As a consequence, significant efforts globally have focused on development, demonstration, and deployment of renewable energy generation sources such as wind, solar photovoltaic, tidal, etc. More recently, the efforts have begun to focus on the deployment of energy storage onto electricity grids.

The variable nature of renewable energy generation can create significant issues with grid stability, demand management, etc. When the intermittent generation is less than 15–20% of the overall energy consumption, grid operators are able to compensate for the effects on grid stability (European Commission, 2013). However, an increase in renewable generation above 20–25% creates significant issues especially when demand is also high when combined with intermittency effects from renewable energy generation (U.S. Energy Information Administration, 2014). To minimize these issues and allow greater penetration of renewable generation into the grid, academia, government, grid operators, regulators, and utilities are recommending storage solutions which can stabilize the grid through a combination of energy shifting or direct smoothing. This brings new opportunities for existing storage technologies. However, since the currently available storage technologies, for example batteries, were not initially designed for such purposes these new applications also bring new science challenges to allow the proven and accepted technologies a new lease in life.

Energy storage integration onto the grid encompasses a range of different applications each with their own unique power, energy, and response time requirements. Furthermore, system size, cycle number, and lifetime requirements also vary for the differing applications. A range of different grid applications where energy storage (from the small kW range up to bulk energy storage in the 100's of MW range) can provide solutions and can be integrated into the grid have been discussed in reference (Akhil et al., 2013). These requirements coupled with the response time and other desired system attributes can create limitations on where the energy storage technologies described above can be effectively used.

**Figure 13:**
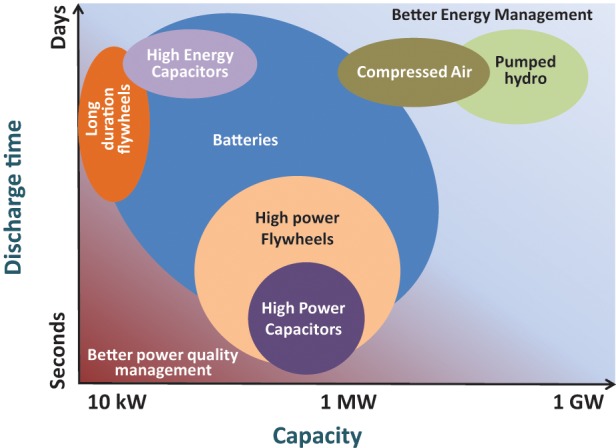
**Approximate representation of characteristics of different storage technologies**. Some types, especially “batteries,” encompass or overlap many technologies within the general shape. Redrawn from the data in Chatzivasileiadi et al. (2013).

Figure 13 shows the types of requirements of storage time, power and response time and the types of applications (Chatzivasileiadi et al., 2013). Differing technologies have different power and energy performance characteristics and therefore the application limitations of different technologies are quite obvious in Figure 13. Clearly based on the data some systems will not be suitable for power quality type of applications whilst other would not be suitable for bulk long-term storage type of applications. The performance characteristics of selected energy storage technologies are described in more detail in Table 3 (Chatzivasileiadi et al., 2013).

**Table 3:** **Characteristics of different battery energy storage technologies are summarized (adapted from Chatzivasileiadi et al., 2013)**.

**Technology**	**Typical lifetime years (cycles)**	**Power density Wkg^−1^/kWm^−3^**	**Energy density Whkg^−1^/kWhm^−3^**	**Typical discharge time**	**Recharge time**	**Response time**	**Operating temperature°C**
Lead acid	3–15 (2000)	75–300/90–700	30–50/75	s-3 h	8–16 h	ms	25
NiCd	15–20 (2500)	150–300/75–700	45–80/<200	s-h	1 h	ms	−40 to 45
Li-ion	8–15 (4 × 10^3^)	230–340/1300–10000	100–250/250–620	min-h	Min-h	ms-s	−10 to 50
NaS	12–20 (>2000)	90–230/120–160	150–240/<400	s-h	9 h	ms	300
Na-NiCl	12–20 (>1000)	130–160/250–270	125/150–200	min-h	6–8 h	ms	270 to 350
Zn-Br_2_ FB	5–10 (>2000)	50–150/1–25	60–80/20–35	s-10 h	4 h	<1 ms	20 to 50
V-Redox FB	10–20 (13 × 10^3^)	NA/0.5–2	75/20–35	s-10 h	Min	<1 ms	0 to 40
Flywheel	>20 (10^7^)	400–1600/5000	5–130/20–80	15 s–15 min	<15 min	ms-s	20 to 40
Super/DL capacitors	>20 (5 × 10^5^)	0.1–10/40000–120000	0.1–15/10–20	ms-1 h	s-min	ms	−40 to 85
Pumped-Hydro	50–100 (>500)	NA/0.1–0.2	0.5–1.5/0.2–2	h-days	Min-h	s-min	Ambient
Compressed air (CAES)	25–40 (No limit)	NA/0.2–0.6	30–60/12	h-days	Min-h	1–15 min	Ambient

From a future technology deployment perspective, different energy storage technologies have a differing level of maturity (International Electrotechnical Commission, 2011). Some technologies are suitable for immediate deployment for grid applications whereas a number of others still require further research and development to improve performance and lifetime and also develop low cost mass production processes before these can be deployed on a large scale. Aside from the technical challenges described above, consideration also needs to be given to the economics and business models for the energy storage deployment. A simple methodology developed by the US DoE can be used to understand the monetary values of different technologies. The method involves taking into consideration the economic factors from location where the storage is to be located, the market, the asset type and who the owner is, then factoring in how the storage system will be used, the benefits and finally calculating a monetary value (Akhil et al., 2013). The methodology needs to be expanded to include modifications to also take account of additional devaluing items such as environmental and installations costs.

The key future requirements and challenges that energy storage technologies face are low installation costs, high durability and reliability, long service lifetimes and high round trip efficiency (U.S. Energy Information Administration, 2014). Furthermore, operation and maintenance costs are also critical in large scale deployment of energy storage solutions for the grid. Clearly, these requirements bring forward the need for scientific advances in the existing technologies which allow either a reduction in manufacturing/materials costs or longer service times etc. Many energy storage solutions which are commercially available have not been designed for large scale deployment, and this is holding these technologies back for grid deployment. Key advances in materials science or engineering as well as process science exist and provide ample opportunities for researchers in the future.

## Membrane separation technologies

The developments in membranes for gas separation have much wider implication in low emission power generation, for controlling gas atmosphere and production of hydrogen and oxygen for a range of applications. In this regard a number of electrochemical gas separation technologies, mostly based on solid electrolytes are under development. All solid state electrochemical cells, where the electrolyte membrane is an oxygen-ion or a proton conductor (pure ionic or mixed ionic/electronic), can be used for selective transport of oxygen or hydrogen in the form of ionic flux thereby acting as electrochemical filters for molecular transport of oxygen or hydrogen.

Apart from the hydrogen production technologies discussed above, there has been a strong emphasis on developing both proton conducting polymer and oxygen-ion conducting ceramic membranes for high purity oxygen production for medical (e.g., home care oxygen therapy), defense, space and clean energy production applications (Badwal and Ciacchi, 2001; Badwal et al., 2003; Phair and Badwal, 2006a; Ursua et al., 2012). For example, in a concept described by Giddey et al. (Ursua et al., 2012), an electrolysis cell based on the proton conducting polymer membrane NAFION was used to split water to produce oxygen on one side of the cell with protons migrating through the membrane to the other electrode/electrolyte interface which then reacted with oxygen from air supplied to produce water. In this mode of operation, one half of the electrochemical cell operated in the water electrolysis mode and the other half in the fuel cell mode thus reducing by 30–40% the power required by a normal water electrolysis cell (Ursua et al., 2012).

The ceramic membranes for high purity oxygen production are based on O^2−^ conducting solid electrolyte such as zirconia, ceria, and bismuth oxide doped with divalent or trivalent cations such as Ca^2+^, Y^3+^, Yb^3+^, Sc^3+^, Gd^3+^, etc. (Badwal and Ciacchi, 2000). Although solid electrolytic cells based on pure ionic conductors are useful for oxygen removal to generate inert atmospheres or for oxygen level control, their use for large scale oxygen production is limited to specific applications (Badwal et al., 2003) due to the large energy input (applied voltage) required to drive across the electrochemical cell. For bulk oxygen production applications such as oxyfuel combustion, mixed ionic/electronic conductors (MIEC) have been considered and technology developed based on such materials (Zhang et al., 2011). These devices typically rely on oxygen partial pressure differential across the MIEC membrane to transport oxygen through the membrane.

In hydrogen production from fossil fuels, hydrogen separation and purification is a key step. The HT ceramic based proton conducting membranes have been considered for pumping hydrogen across an electrochemical cell (Phair and Badwal, 2006b; Gallucci et al., 2013). The use of pure ionic conducting membranes is energy intensive as these devices are driven by external voltage or current. However, mixed proton/electronic conducting membranes are of interest for separating hydrogen for example from a mixture of CO_2_ and H_2_ following gasification of coal or reforming of NG. Recent reviews discuss many proton conducting membrane materials and gas separation reactors (Phair and Badwal, 2006b; Gallucci et al., 2013).

In the area of gas separation membranes, there are major technical challenges in fabrication of composite structures, chemical and thermal compatibility between components of the composite structure, interface coherency, optimization of the microstructure, lifetime issues in real operating environments (integrated into coal gasification, NG reforming plants), fabrication of support structures for deposition of thin films of the membrane material with optimal properties to achieve desired hydrogen or oxygen permeation rates and selectivity to the transporting specie. Some of the other major issues are related to fabrication, up-scaling and to have good mechanical strength and toughness as well as good chemical stability in real operating environments.

## Electrochemical reactors for energy conversion and storage

Interest in electrochemical reactors stem from the fact that energy can be converted from one form to another more useful form for easy storage and transportation (for example, hydrogen, ammonia, or syn gas—a precursor for the liquid fuel production—with the use of a renewable energy source). In electrochemical cells, electrochemical processes can also be used to produce value added fuels or chemicals. Several different types of systems based on liquid and solid electrolytes have been proposed. The major advantage of the solid electrolyte systems is that both reactant and product chemicals are separated by the electrolyte membrane and a wide range of operating conditions are available to suit a particular chemical/electrochemical reaction. Two types of systems under development are based on oxygen-ion or proton conducting electrolytes. The selectivity to partial oxidation/reduction reaction can be controlled by the suitable choice of catalytic electrodes or catalyst/electrode mixtures, by the careful control over migration rates of oxygen-ion or protons and cell operating conditions. In the three sections below some electrochemical processes are briefly described.

### Partial oxidation, hydrogenation, dehydrogenation reactors

**Figure 14:**
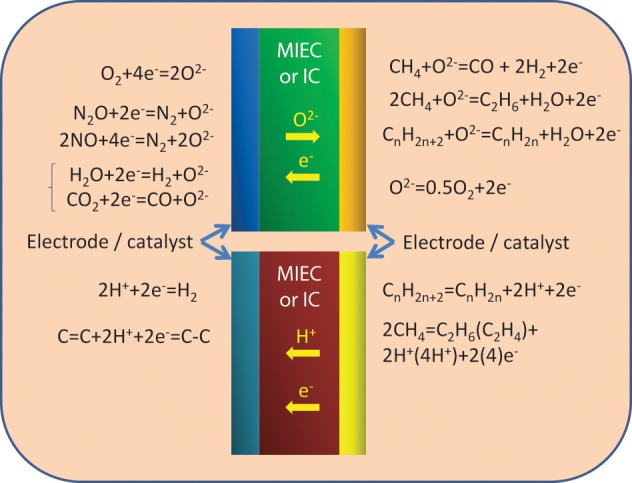
**Basic operating principle of O^2-^ and H^+^ electrochemical reactors for fuel and chemical production**.

In these reactors either a pure O^2−^ or H^+^ conducting (IC) or a mixed O^2−^/e^−^ or H^+^/e^−^ conducting (MIEC) membrane is used to separate the reactant and products (Iwahara et al., 2004; Sundmacher et al., 2005; Wei et al., 2013). These materials have typically perovskite (ABO_3_), fluorite (MO_2_), or pyrochlore (A_2_B_2_O_7_) structures. Often electrode/catalyst layer is applied to both sides of the ion conducting membrane as shown in Figure 14. The O^2−^ or H^+^ ions migrate under an applied electric field or partial pressure differential of the migrating specie and either oxidize or reduce the reactant to produce fuels or value added chemicals.

Dense ceramic membranes with mixed O^2−^/e^−^ have been used for the conversion of methane to syngas, oxidative coupling of methane to higher hydrocarbons (C_2_), conversion of ethylbenzene to styrene, and oxidative dehydrogenation of alkanes to olefins and conversion of pollutants such as N_2_O and NO to N_2_ by extracting oxygen (Wei et al., 2013). Similarly proton conducting membranes can be used for hydrogenation or dehydrogenation reaction by adding or stripping hydrogen from organic compounds (CH_4_ to C_2_H_4_, C_2_H_6_; C–C to C=C; C=C to C–C) (Sundmacher et al., 2005; Wei et al., 2013) (Figure 14). Various processes, electrochemical reactors and materials of construction based on dense O^2−^ and H^+^ conducting ceramic membranes have been reviewed extensively in the literature (Iwahara et al., 2004; Sundmacher et al., 2005; Wei et al., 2013).

There are a number of material, fabrication, design and up-scaling challenges for a given type of electrochemical reactor. Often materials are exposed to strongly oxidizing or reducing conditions at HTs. This chemical stability and thermal compatibility of all cell components needs to be addressed. The selectivity to a particular reaction and production rates often compete and for given reaction conditions undesirable products can easily form. Apart from the general criteria of high ionic flux for the transporting specie and thermal and chemical stability of the membrane materials, for the type of electrochemical reaction to take place, several materials and operating conditions need to be optimized.

### Waste to fuels and value added products

The electrochemical conversion of waste products such as biomass (agricultural and forest residue), municipality, or industrial waste to value added chemicals and fuels is an area of enormous interest globally from the commercial as well as environmental view point. These waste materials can be converted to electricity, heat, gaseous (CO, H_2_, CH_4_), or liquid fuels (methanol, ethanol, biodiesel, etc.) by employing HT processes which are highly efficient and CO_2_ neutral.

#### Microbial electrochemical system for hydrogen and biofuel production

One of the rapidly developing areas for conversion of waste to value added chemicals is based on a microbial electrochemical system called microbial electrolysis (Logan and Rabaey, 2012; Wang and Ren, 2013). In a microbial electrolysis cell (MEC), the organic and inorganic parts of the waste material in the anode chamber of the cell are oxidized with the help of microorganisms (electrochemically active bacteria) to CO_2_ and electrons. The electrons are passed on to the electrode, and protons thus generated are transported through the electrolyte. In the cathode chamber, the protons can either react with electrons supplied from the external circuit to produce hydrogen (as a fuel) or can be made to react (hydrogenation) with another species to produce other value added chemicals such as biofuels.

**Figure 15:**
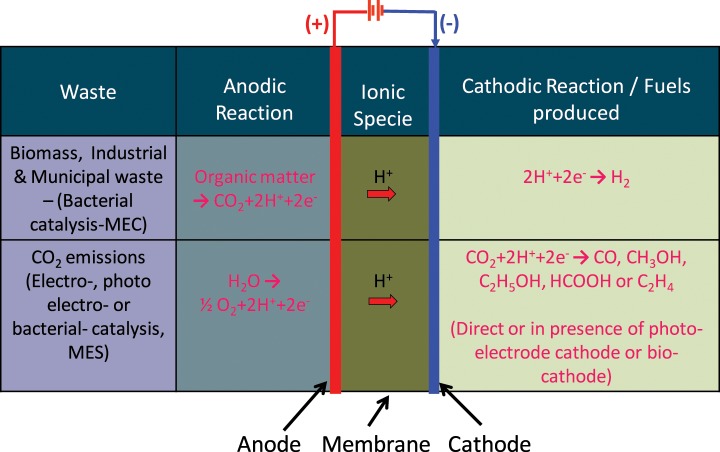
**Electrochemical reactions involved in various processes for producing fuels and value-added chemicals from waste**.

Figure 15 illustrates this process schematically. The theoretical voltage required for producing hydrogen by MEC is 0.41 V compared to 1.2 V for conventional water electrolysis, however, applied voltages as high as 1 V is required for MEC to achieve practical hydrogen generation rates (Logan and Rabaey, 2012). By employing renewable and waste materials in MEC, the hydrogen production rates of more than three times have been achieved compared to those obtained by dark fermentation (Wang and Ren, 2013). The major challenge for commercialization of this technology is the cost of precious metal catalyst electrodes and other associated materials (Logan and Rabaey, 2012), and the sluggish reaction rates to achieve practical hydrogen or other chemical production rates.

#### Conversion of CO_2_ to fuels with renewable energy

Another emerging area under development energy conversion and storage involves the utilization of CO_2_ as the feedstock to electrochemically synthesize fuels and certain specialty chemicals such as carbon monoxide, methanol, formic acid, methane, ethylene, and oxalic acid (Jitaru, 2007). The utilization of electricity from renewable sources to convert CO_2_ to high energy density fuels can help in alleviating the challenges of intermittent nature of the renewable sources by storing energy in the form of high energy density fuels, as well as addressing the liquid fuel shortage for the transport sector. Apart from the production of fuels, some products formed by CO_2_ conversion may also be suitable as a feedstock for the chemical, pharmaceutical, and polymer industries. A number of review articles provide details on the methods of CO_2_ reduction, electrode/electrolyte systems under consideration, various chemical products that can be produced and current status of the technology (Jitaru, 2007; Hori, 2008; Lee et al., 2009; Beck et al., 2010; Li, 2010; Whipple and Kenis, 2010; Hu et al., 2013; Jhong et al., 2013; Qiao et al., 2014). The processes employed for the electrochemically conversion of CO_2_ include electro-catalysis (direct electrochemical conversion), photo electro-catalysis and bacteria-assisted electro-catalysis as shown schematically in Figures 14, 15. Although many processes are at an early stage of technological developments and there are concerns about the economic viability, these processes are discussed briefly in the following sections.

***Direct electrochemical conversion***. The main electrolyte systems under consideration for the direct electrochemical conversion of CO_2_ are divided into low or ambient temperature systems [aqueous, non-aqueous (Cook et al., 1990; Hara et al., 1995; Hara and Sakata, 1997; Jitaru, 2007; Ogura, 2013) and PEM-based (Delacourt et al., 2008; Aeshala, 2013) electrolytes]; and HT systems [molten carbonate (Licht et al., 2010) and solid oxide (Stoots, 2006, 2010; Bidrawn et al., 2008; Hartvigsen et al., 2008; Ebbesen and Mogensen, 2009; Zhan et al., 2009; Fu et al., 2011; Graves et al., 2011; Narasimhaiah and Janardhanan, 2013) electrolytes—in the 700–1000°C range]. In the direct electro-catalysis process, CO_2_ is supplied as a feedstock to the cathode chamber of the cell for reduction. In case of LT electrolyte systems (aqueous and PEM electrolytes), water is supplied to the anode as a source of protons for reaction at the cathode (Delacourt et al., 2008; Aeshala, 2013; Ogura, 2013). The protons transported through the electrolyte to the cathode are made to react with CO_2_ to produce fuels or chemicals (Figures 14, 15). The competing reaction in aqueous- and PEM-based electrolytes is the hydrogen evolution that should be avoided, otherwise it results in wastage of energy input to the process if hydrogen is not the required chemical. Most metallic electrodes employed in the process yield CO and HCOOH, however, copper can also yield hydrocarbons such as methane and ethylene (Jitaru, 2007). Ogura has recently reported the CO_2_ reduction to ethylene on copper halide confined copper mesh electrode with current efficiency of up to 80% and selectivity of up to 87% (Ogura, 2013).

In a molten carbonate electrolyte system, CO_2_ is dissolved in the carbonate bath and is reduced to CO via the electrolysis process. The electrical energy input for the endothermic CO_2_ reduction reaction reduces as the process is carried out at HTs with solar thermal energy input (Licht et al., 2010). In a solid oxide electrolyte system, CO_2_ supplied to the cathode is reduced to CO and oxygen anions thus formed are transported through the solid electrolyte to produce oxygen at the anode. The solid oxide electrolyte cells have also been investigated for co-electrolysis of CO_2_ and water (Figure 14). In this case, steam and CO_2_ are both supplied to the cathode that results in formation of syn gas (H_2_ + CO) at the cathode and oxygen at the anode (Stoots, 2006; Hartvigsen et al., 2008). Although the electrochemical conversion of CO_2_ to different hydrocarbon fuels has been demonstrated by a number of investigators, the real challenges are to improve the conversion rates (CO_2_ being a stable molecule and is difficult to reduce) and energy efficiencies to make the process commercially viable. Thus new catalysts, processes and materials need to be developed to reduce cell voltage losses and improve the selectivity and conversion efficiency (Whipple and Kenis, 2010; Hu et al., 2013). In a recent article, Jhong et al. have covered the current status, challenges, and future opportunities for electrochemical conversion of CO_2_ to useful chemicals (Jhong et al., 2013).

***Photo electrochemical conversion***. In a photo electro-catalysis process, a photo-reduction electrode that consists of a semiconductor and a photo-catalyst is used as a cathode (Hu et al., 2013). The photons from the solar radiation, absorbed by the semiconductor cause the excited electrons transfer from valence to conduction band, that results in transfer of electrons to photo-catalysts. This electron transfer assists in the CO_2_ reduction reaction involving protons transported through the electrolyte to produce CO and other organic compounds (Figure 15). It has been reported that the onset voltages for the CO_2_ reduction process are significantly reduced by employing photo electrodes (cathode) compared to metallic electrodes (Kumar et al., 2012; Hu et al., 2013). Both aqueous and non-aqueous systems have been explored for the photo electrochemical reduction of CO_2_. Higher solubility of CO_2_ in non-aqueous electrolytes compared to aqueous electrolytes is favorable to achieve high current densities and increase selectivity over hydrogen evolution, however, other means such as high pressure and employing gas diffusion electrodes can be used for both types of electrolytes to increase CO_2_ concentration. Some of the electrode/electrolyte systems investigated for the CO_2_ reduction in aqueous media are p-Si / NaSO_4_, p-CdTe and p-InP/tetraalkylammonium, p-GaAs in KCl, HClO_4_, or Na_2_CO_3_ electrolyte (Barton et al., 2008; Kumar et al., 2012). Other photo electrodes explored for CO_2_ reduction are Cu, Ag or Au, Pd nano particles attached to p-Si or p-InP (Barton et al., 2008; Kumar et al., 2012). Although the photo electrodes investigated for the non-aqueous electrolytes have been same as for aqueous electrolytes, the popular electrolyte used has been methanol, due to its high CO_2_ solubility. The chemicals produced, and the Faradaic efficiency and selectivity of the chemical produced depends on the photo electrode and the supporting electrolyte used. These systems have been reviewed quite extensively by Kumar et al. (2012) and more details on performance of these systems can be found in this review. The low efficiencies and current densities achieved, and the high costs of the catalysts used in this process are still some of the major challenges for this technology.

***Bacterial-assisted electrochemical conversion***. In bacteria-assisted electrosynthesis, the microorganisms at the cathode of the electrochemical cell assist in the reduction of CO_2_ to fuels or value added chemicals. This process is also called microbial electrosynthesis (MES) (Wang and Ren, 2013). As depicted in Figure 15, the process involves protons transported through the electrolyte, electrons delivered to cathode and CO_2_ supplied to the cathode camber. It is claimed that with electric input from renewable energy sources, the microbes can harvest the solar energy at 100 times the efficiency of a biomass-based fuel/chemical production (Wang and Ren, 2013). The formation of products that have already been demonstrated from this route by employing various types of cultures, are methane, acetate, and oxo-butyrate. In another variation to the MEC, described in Section Microbial Electrochemical System for Hydrogen and Biofuel Production, if the protons transported through the electrolyte to cathode (biocathode) are made to react with the CO_2_, other chemicals can be formed in preference to hydrogen generation. In a recent study employing a MEC based on a cation exchange membrane, CO_2_ was successfully converted to methane for a period of 188 days with an overall energy efficiency of 3.1% (Van Eerten-Jansen et al., 2011). The rates and quantities of the chemical produced by microbial synthesis and electrolysis cells, and the overall energy efficiencies are very low, and would require significant improvements to the synthesis process as well as the cell configuration to lower resistive losses in the various cell components for a large scale operation (Van Eerten-Jansen et al., 2011; Logan and Rabaey, 2012).

### Electrochemical processes for ammonia production

Ammonia is an excellent energy storage media with infrastructure for its transportation and distribution already in place in many countries. Liquid ammonia has a hydrogen content of 17.6 wt% and therefore can be utilized as a source of hydrogen at distributed sites. By comparison the hydrogen content in methanol is only 12.5 wt%. Over 200 million metric tons of ammonia is produced per annum globally and in terms of production volumes, it is one of the major chemicals produced. Current ammonia production processes are highly energy intensive (Giddey et al., 2013). Ammonia is the intermediate chemical for the production of many chemicals including over 80% utilization for fertilizer production with other important uses including the manufacture of explosives, pharmaceutical chemicals and other industrial processes such as synthesis of specialty ceramic powders and refrigeration.

Ammonia is produced at present through the well-known Haber-Bosch process. Given the high energy consumption and high capital cost of the process requiring hydrogen and nitrogen to react on an iron-based catalyst at HTs (up to 550°C) and high pressures (up to 300 bar). In view of this a number of alternative processes are under investigation. Amongst many approaches, electrochemical routes have the potential to produce ammonia under very mild conditions of temperature and pressure and at a lower cost compared with the Haber-Bosch process of ammonia production (Giddey et al., 2013).

**Figure 16:**
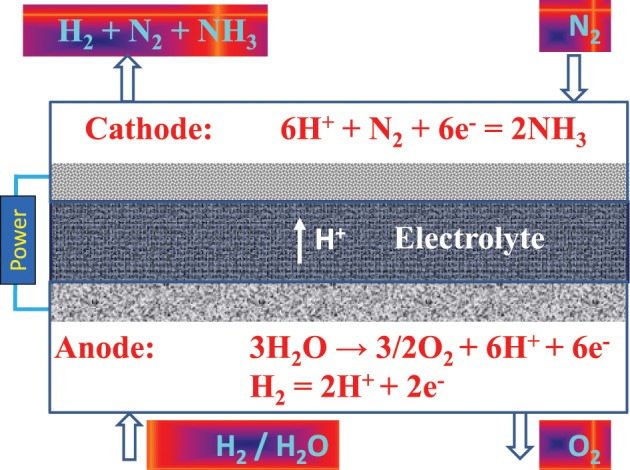
**The operating principle of ammonia production in a solid state electrochemical cell**.

The various electrochemical routes for ammonia production are differentiated by the type of electrolyte used and the operating temperature regime. These can be broadly divided into four categories: (a) liquid electrolytes operating near room temperature, (b) molten salt electrolytes operating in the 300–500°C range, (c) composite electrolytes consisting of a solid and a low melting molten salt, and (d) solid electrolytes with operating temperature range from room temperature (typically polymers) to 800°C (ceramics). Other materials of construction are based on the type of system selected. Typical operation of an electrochemical ammonia production process is described in Figure 16. These systems have been discussed in detail in recent reviewed articles (Amar et al., 2011; Giddey et al., 2013; Garagounis et al., 2014) and involve supply of hydrogen at the anode/electrolyte interface, migration of protons through the electrolyte and reaction with N_2_ over a cathode catalyst to form ammonia. Materials requirements include high ionic conductivity in the electrolyte and chemical stability under operating conditions and thermo-mechanical compatibility between various cell components. The catalyst on the nitrogen side plays a critical role.

Two critical performance parameters that determine the overall process efficiency are the current efficiency and ammonia production rates. The current efficiency or conversion rates determine the percentage of protons flowing through the electrolyte that are effectively utilized in ammonia formation. The ammonia production rates are defined in number of moles of ammonia produced per unit cell area per unit time typically expressed as mol.cm^−2^.s^−1^. Both high ammonia production rates and high current efficiency are essential for the economic viability of the process. The higher operating temperature improves kinetics of reaction between nitrogen and hydrogen and would allow integration with thermal solar or nuclear power plants for heat input. However, the thermodynamics of the reaction favors operation at LTs and high pressures and hence offer the potential to use low cost materials.

This technology is at an early stage of development requiring considerable work on the development of cell materials and ammonia production catalyst. The ammonia production rates achieved by various electrochemical processes are in the 10^−13–10^−8^^ mol.cm^−2^.s^−1^ and are too low for the process to be economically viable. The highest production rate reported was for a PEM-based electrochemical reactor. Often high production rates are quoted at low current densities and for high hydrogen conversion rates (over 50%) reported in the literature, the ammonia production rates are low (Giddey et al., 2013). At least another order of magnitude increase in ammonia production rates with conversion efficiency well above 50% at current densities above 0.25 A.cm^−2^ would make the process technically feasible for consideration of the technology for commercialization. Lifetime, degradation rates, cost of materials and fabrication processes, and up-scaling are some of the other considerations.

## Conclusion

Electrochemical energy technologies are already contributing substantially to reduction of pollution and greenhouse gas emissions, in process control and via increasing energy conversion efficiency. The growing demand for technologies that can stabilize power generation and delivery is driving research toward developing new technologies. This is increasing the number of systems under investigation across the entire innovation chain from very early stage research through to development of conventional devices to increase performance and reduce cost. As with all new technologies there remain many technical challenges facing the developers of future electrochemical power systems, however, the increased understanding of the value of these technologies is leading to an increase in the scale of programs looking to improve these technologies. It is unclear which new technologies will emerge as leaders in the future power market but it is clear that there will be significant improvement over current devices in terms of cost reduction, performance, and availability over the next decade. This will go beyond lone new electrochemical cell chemistries and will increasingly involve the development of highly integrated hybrid systems that take advantage of the strengths of multiple technology features.

### Conflict of interest statement

The authors declare that the research was conducted in the absence of any commercial or financial relationships that could be construed as a potential conflict of interest.
